# Superparamagnetic iron oxide nanoparticle-driven 3-aroyl-1,4-diarylpyrrole nanocomposites (ARDAP@SPION-PEI) mediate renal cancer cell PANoptosis by regulating chromatin accessibility

**DOI:** 10.1186/s43556-026-00470-z

**Published:** 2026-05-15

**Authors:** Hongliang Shen, Zeyu Cui, Yilun Wu, Michela Puxeddu, Yanchen Lai, Ren Mo, Boyu Yang, Yinong Niu, Yichao Wen, Xiling Du, Romano Silvestri, Te Liu

**Affiliations:** 1 Department of Urology, Beijing Friendship Hospital, Capital Medical University, 95 Yongan Road, Beijing, 100050 China; 2 Institute of Urology, Beijing Municipal Health Commission, Beijing, 100050 China; 3 Shanghai Geriatric Institute of Chinese Medicine, Shanghai University of Traditional Chinese Medicine, 365 South Xiangyang Road, Shanghai, 200031 China; 4 College of Biotechnology and Pharmaceutical Engineering, Nanjing Tech University, Nanjing, 211816 China; 5 Department of Drug Chemistry and Technologies, Sapienza University of Rome, Piazzale Aldo Moro 5, 00185 Rome, Italy; 6 Department of Urology, Inner Mongolia People’s Hospital, Inner Mongolia Urological Institute, Hohhot, Inner Mongolia 010017 China; 7 School of Life Science and Technology, Tongji University, Shanghai, 200092 China

**Keywords:** Renal cancer stem cells, Superparamagnetic iron oxide nanoparticles, 3-Aroyl-1,4-diarylpyrrole, PANoptosis, Chromatin accessibility, ZNF148

## Abstract

The renal cancer stem cells (RCSCs) have highly proliferation, resistance to radiotherapy and chemotherapy, and enhanced invasive and tumorigenic capacities characteristics. 3-Aroyl-1,4-diarylpyrrole (ARDAP) is a potent microtubule inhibitor developed by our team; however, its specific killing effects on RCSCs remain unknown. Furthermore, clinical application of ARDAP is restricted by shortcomings such as poor water solubility and low utilization rates. Here, we developed polyethylenimine-coated superparamagnetic iron oxide nanoparticles loaded with ARDAP (ARDAP@SPION-PEI) to overcome these barriers and evaluated their efficacy in mouse RCSCs (mRCSCs) and zebrafish. In vitro and in vivo experiments demonstrated that ARDAP@SPION-PEI significantly inhibited mRCSCs proliferation, migration, and tumorigenicity while suppressing angiogenesis in zebrafish. ARDAP@SPION-PEI treatment significantly upregulated the expression of key PANoptosis pathway genes. Assay for transposase-accessible chromatin using sequencing revealed widespread chromatin accessibility alterations, with prominent enrichment of the transcription factor zinc finger protein 148 (ZFP148). Luciferase reporter assays demonstrated that ZFP148 directly bound to and activated promoters of key PANoptosis-related genes, including receptor-interacting serine/threonine kinase 3 (*Ripk3*), gasdermin D (*Gsdmd*), IL-1β (*Il1b*), and caspase-1 (*Casp1*). Chromatin immunoprecipitation–polymerase chain reaction further showed significantly enhanced promoter occupancy at the histone H3 lysine 4 trimethylation site following treatment, indicating transcriptionally active chromatin states. Silencing *ZFP148* attenuated gene activation and reduced cell death induction. In conclusion, ARDAP@SPION-PEI induces mRCSC PANoptosis by remodeling chromatin structure to promote ZNF148-mediated transcription, offering a promising epigenetic-based nanotherapeutic strategy for renal cancer.

**Supplementary Information:**

The online version contains supplementary material available at 10.1186/s43556-026-00470-z.

## Background

Renal cell carcinoma (RCC), including renal adenocarcinoma, is a malignant tumor arising from the renal parenchyma and is associated with poor patient survival [1–3]. Its pathogenesis is complex and not yet fully elucidated, but it is believed to involve multiple factors, including genetic predisposition, smoking, obesity, hypertension, and antihypertensive medication use [1–3]. Increasing evidence indicates that renal cancer tissues harbor a small yet highly proliferative subpopulation known as renal cancer stem cells (RCSCs), which exhibit resistance to radiotherapy and chemotherapy, enhanced invasive and tumorigenic capacities, and expression of embryonic stem cell markers [1, 4–8]. Commonly recognized biomarkers of cancer stem cells include CD44, CD133, c-Kit (CD117), Sox2, and Oct-4 [4–7]. However, cancer stem cells derived from different tissues not only express these shared markers but also display lineage-specific surface phenotypes [4, 5, 7, 8]. For example, breast cancer stem cells are characterized by CD44 + /CD24 − /low, intestinal cancer stem cells by CD44 + /Lgr5 + , ovarian cancer stem cells by CD44 + /CD133 + or CD44 + /c-Kit + , prostate cancer stem cells by CD44 + /ABCG2 + , and acute myeloid leukemia stem cells by CD133 + /CD34 + /CD38 − [4–7]. RCSCs are typically defined by the CD44 + /CD105 + phenotype [1, 3, 4, 7, 9]. Owing to their intrinsic resistance to conventional therapies and strong tumor-initiating capacity, RCSCs are considered major drivers of renal cancer metastasis and recurrence [1, 4–8].

Although several microtubule-targeting chemotherapeutic agents, including paclitaxel, docetaxel, and vincristine, are widely used in cancer treatment, cancer stem cells frequently exhibit substantial resistance to these conventional agents. Therefore, the development of novel microtubule inhibitors capable of effectively targeting cancer stem cells remains an urgent clinical need. 3-Aroyl-1,4-diarylpyrrole (ARDAP) is a newly developed and highly potent microtubule inhibitor generated by our group [10–14]. ARDAP suppresses microtubule assembly and polymerization in cancer cells by binding to the colchicine site of tubulin [10–14]. Previous studies have demonstrated that ARDAP and its derivatives induce G2/M-phase cell-cycle arrest and mitochondrial-dependent apoptosis in chronic myeloid leukemia cells, thereby markedly inhibiting cell proliferation [10–14]. Furthermore, we have shown that ARDAP significantly suppresses tumor formation and growth in ovarian clear cell carcinoma and bladder cancer xenograft models by impairing tumor angiogenesis and disrupting mitochondrial integrity [11, 14]. However, whether ARDAP can effectively eliminate renal cancer stem cells remains unknown. In addition, its poor aqueous solubility, limited dispersibility, and low bioavailability considerably restrict its clinical translation.

Superparamagnetic iron oxide nanoparticles (SPIONs) have attracted considerable attention in gene delivery research because of their controllable physicochemical properties, high stability, and ease of surface modification [15–18]. Upon complexation with plasmid DNA or noncoding RNA, SPIONs can facilitate nucleic acid transfection into mammalian cells under an external magnetic field. Through magnetic guidance and adsorption, they can overcome intracellular and extracellular barriers, increase local nucleic acid concentrations, and enhance transfection efficiency [15, 19–23]. Moreover, surface functionalization with cationic polymers, such as polyethyleneimine, dendrimers, glucose, chitosan, or cationic liposomes, further strengthens interactions between nanomaterials and nucleic acids, thereby improving delivery efficiency [16, 18, 23, 24]. Beyond gene delivery, SPIONs can also serve as nanocarrier systems for chemotherapeutic agents, improving drug solubility, dispersibility in aqueous environments, bioavailability, and cytotoxic efficacy against tumor cells [19, 22–25]. These advantages highlight the substantial translational potential of SPION-based nanotherapeutics. However, to date, no studies have investigated the combination of 3-aroyl-1,4-diarylpyrrole with SPION-based delivery systems.

PANoptosis is an inflammatory programmed cell death pathway regulated by the PANoptosome complex and integrates key molecular features of pyroptosis, apoptosis, and necroptosis, rather than representing any single pathway alone [26–29]. The term “PANoptosis” was first introduced in 2019 by Malireddi et al., who demonstrated that innate immune sensors such as Z-DNA binding protein 1 (ZBP1) and transforming growth factor-β–activated kinase 1 (TAK1) play essential roles in PANoptosome assembly [30]. Several molecules, including TAK1, PSTPIP2, SHARPIN, HOIP, HOIL-1, and A20, function as negative regulators of PANoptosome activation [26–29]. Although initially characterized in infectious diseases, accumulating evidence suggests that PANoptosis may have therapeutic relevance in cancer [26–30]. For example, cysteine desulfurase deficiency synergized with oxaliplatin to trigger PANoptosis through reactive oxygen species accumulation [31]. ZBP1-mediated inflammatory cell death has been shown to suppress tumorigenesis in colorectal cancer and melanoma [32, 33]. In breast cancer, transcriptomic analyses revealed that elevated expression of PANoptosis-related genes correlates with reduced tumor incidence [34, 35]. However, whether microtubule-targeting agents can induce PANoptosis in cancer stem cells remains largely unexplored.

In this study, we constructed polyethylenimine (PEI)-coated SPION (SPION-PEI)-driven 3-aroyl-1,4-diarylpyrrole nanocomposites (ARDAP@SPION) and demonstrated that they induce PANoptosis in renal cancer stem cells, as evidenced by suppressed proliferation, migration, and angiogenesis in vitro and in vivo. Considering that PANoptosis requires coordinated transcriptional activation of multiple genes and that gene transcription is tightly regulated by chromatin structure [36], we further investigated changes in chromatin accessibility associated with treatment. Using assay for transposase-accessible chromatin with sequencing, we analyzed genome-wide chromatin remodeling and explored the epigenetic mechanisms underlying PANoptosis induction [36].

## Results

### ARDAP@SPION-PEI significantly inhibit mRCSC activity in vitro

**Fig. 1:**
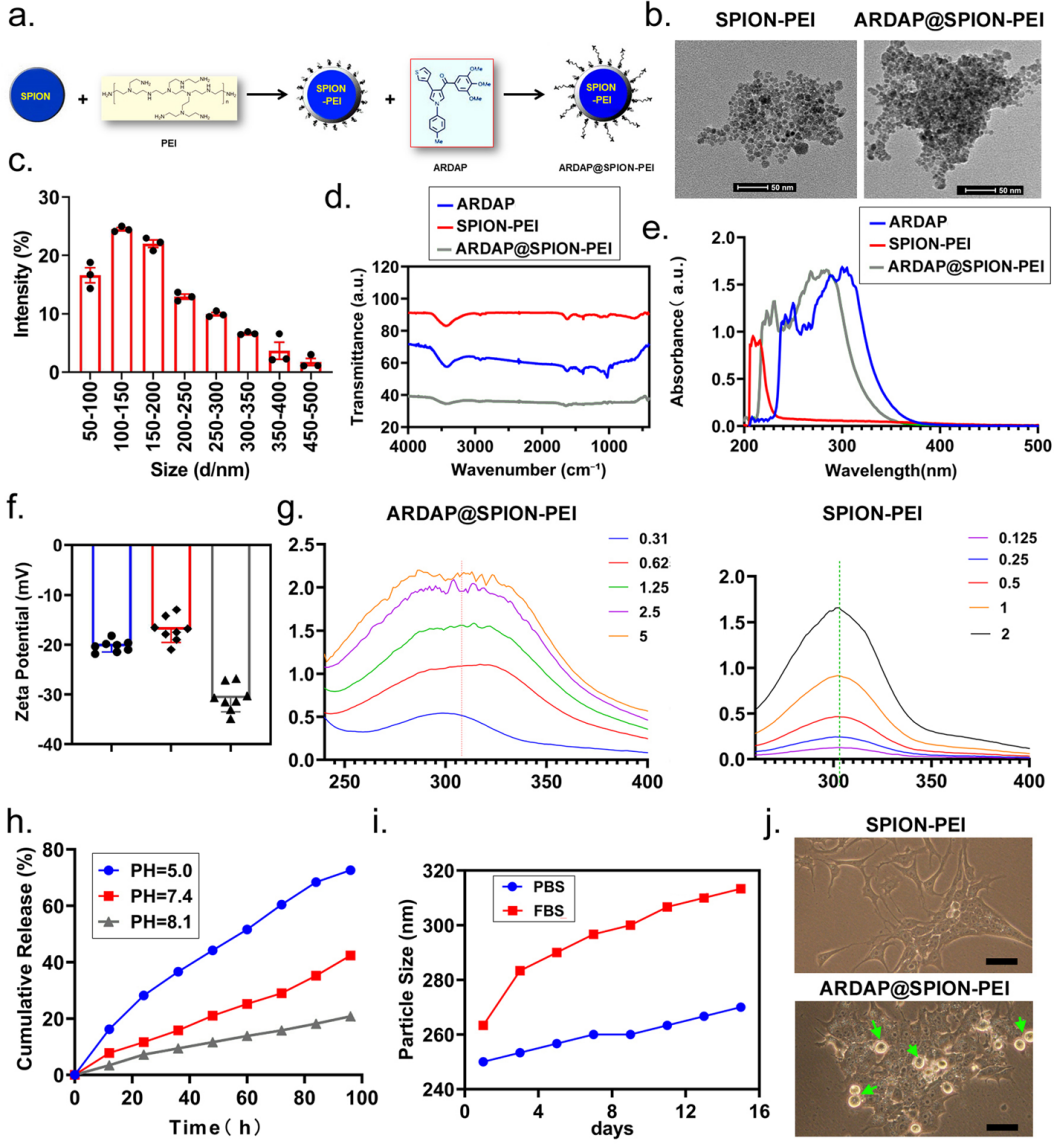
ARDAP@SPION-PEI were synthesized and their physicochemical properties were characterized. **a** Schematic illustration of the synthetic route of ARDAP@SPION-PEI. **b** TEM images showing spherical ARDAP@SPION-PEI particles with sheet-like aggregation (scale bar = 50 nm). **c** The results of NTA showed that the size of ARDAP@SPION-PEI was between 50 and 300 nm (*n* = 3). **d** The results of FT-IR analysis. **e** The UV spectrophotometer detection results. **f** The results of Zeta potential detection indicated that of ARDAP@SPION-PEI zeta potential was about -30.77 ± 2.728 mV (*n* = 8). **g** The packaging closure rate of ARDAP in ARDAP@SPION-PEI was more than 60%. **h** The experimental results showed that the release rate of ARDAP and acidic conditions was directly proportional, and the release rate increased with the elevated of acidity. **i** the particle size of ARDAP@SPION-PEI exposed to serum (FBS) was elevated more quickly, compared to it exposed to PBS. **j** Representative images showing RENCA cell death following ARDAP@SPION-PEI treatment (magnification, 400 × ; scale bar = 15 µm); green arrows indicate dead cells

FT-IR, TEM, and nanoparticle tracking analysis were performed to evaluate the successful loading and physicochemical characteristics of ARDAP@SPION-PEI, including drug loading efficiency, encapsulation efficiency, in vitro release kinetics, and colloidal stability under physiological conditions. Surface coating of SPIONs with PEI facilitated ARDAP adsorption, resulting in the successful formation of ARDAP@SPION-PEI nanocomposites (Fig. 1a). TEM imaging showed that individual ARDAP@SPION-PEI particles appeared as spherical structures, while multiple particles exhibited sheet-like aggregation (Fig. 1b). The results of Nanoparticle tracking analysis (NTA) showed that the size of ARDAP@SPION-PEI nanoparticles was between 50 and 300 nm (Fig. 1c). Fourier transform infrared spectroscopy (FT-IR) analysis revealed that ARDAP and ARDAP@SPION-PEI had a same vibration peak between 1000–2000 nm which confirmed that the ARDAP was loaded successfully onto the SPIONs nanoparticles (Fig. 1d). The UV spectrophotometer detection results indicated that there were two absorption mainly peak of ARDAP between 220–240 nm and 260–300 nm. And, two absorption mainly peak of ARDAP@SPION-PEI were between 240–260 nm and 280–330 nm. So, ARDAP@SPION-PEI and ARDAP intersected at absorption mainly peak values between 280–300 nm (Fig. 1e). The results of Zeta potential detection indicated that of ARDAP@SPION-PEI zeta potential was about -30.77 ± 2.728 mV (Fig. 1f). Besides, the UV visible spectrophotometer detection results showed that the packaging closure rate of ARDAP in ARDAP@SPION-PEI was more than 60% (Fig. 1g). Measuring the release properties of ARDAP@SPION-PEI in different acid/base (different pH value) environments helps us evaluate the controlled release ability and characteristics of nanoparticles drug delivery system. The experimental results showed that the release rate of ARDAP and acidic conditions was directly proportional, and the release rate increased with the elevated of acidity (pH = 5.0 > 7.4 > 8.1). This feature enabled ARDAP@SPION-PEI to better exert its anti-tumor effect (Fig. 1h). In addition, the particle size of ARDAP@SPION-PEI exposed to serum (FBS) was elevated more quickly, compared to it exposed to PBS (Fig. 1i). The results of the above experiments revealed that the ARDAP@SPION-PEI has higher dispersibility and stability characteristics than sole ARDAP. Following exposure to ARDAP@SPION-PEI, RENCA cells displayed marked morphological changes, including increased numbers of rounded, detached, and floating dead cells, indicating pronounced cytotoxic activity (Fig. 1j).

**Fig. 2:**
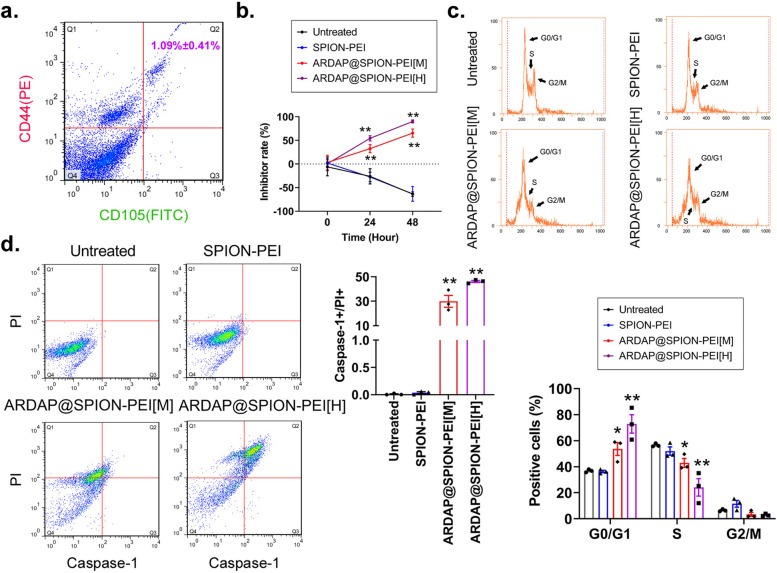
ARDAP@SPION-PEI exposure significantly inhibited cell-cycle progression and in vitro proliferation in mRCSCs. **a** Flow cytometric separation and enrichment of CD44 + /CD105 + mRCSCs from RENCA cells (*n* = 3). **b** CCK-8 assay demonstrating that ARDAP@SPION-PEI significantly suppressed mRCSC proliferation in vitro. **P < 0.01 vs. SPION-PEI, Student’s *t* test (*n* = 3). **c** Flow cytometric analysis showing G0/G1-phase arrest induced by ARDAP@SPION-PEI in mRCSCs. **P < 0.01 vs. SPION-PEI, Student’s *t* test (*n* = 3). **d** Flow cytometric quantification of CASP1 + /PI + cells indicating enhanced inflammatory cell death following ARDAP@SPION-PEI treatment. **P < 0.01 vs. SPION-PEI; *P < 0.05 vs. SPION-PEI, Student’s *t* test (*n* = 3)

To further assess cytotoxicity in mouse renal CSCs (mRCSCs), CD44 + /CD105 + cells were isolated and enriched from RENCA cells using flow cytometry (Fig. 2a). Consistent with stem cell characteristics, CD44 + /CD105 + mRCSCs exhibited enhanced sphere-forming capacity, elevated expression of “stemness”-associated markers, and greater resistance to conventional chemotherapeutic agents compared with CD44 − /CD105 − cells (Fig. S1). CCK-8 assays demonstrated that treatment with ARDAP@SPION-PEI[M] or ARDAP@SPION-PEI[H] for 24 h significantly increased the proliferation inhibition rate relative to the SPION control group in a dose-dependent manner (Fig. 2b). Importantly, ARDAP@SPION-PEI[M] did not significantly affect the proliferation of murine mesenchymal stem cells in vitro (Fig. S2), suggesting selective cytotoxicity toward cancer stem cells. Cell cycle analysis revealed that ARDAP@SPION-PEI treatment significantly increased the proportion of cells in the G0/G1 phase while reducing the S-phase population, indicating induction of G0/G1 arrest (Fig. 2c). Furthermore, treatment markedly elevated the percentage of Caspase-1 + /PI + cells, consistent with inflammatory cell death (Fig. 2d).

**Fig. 3:**
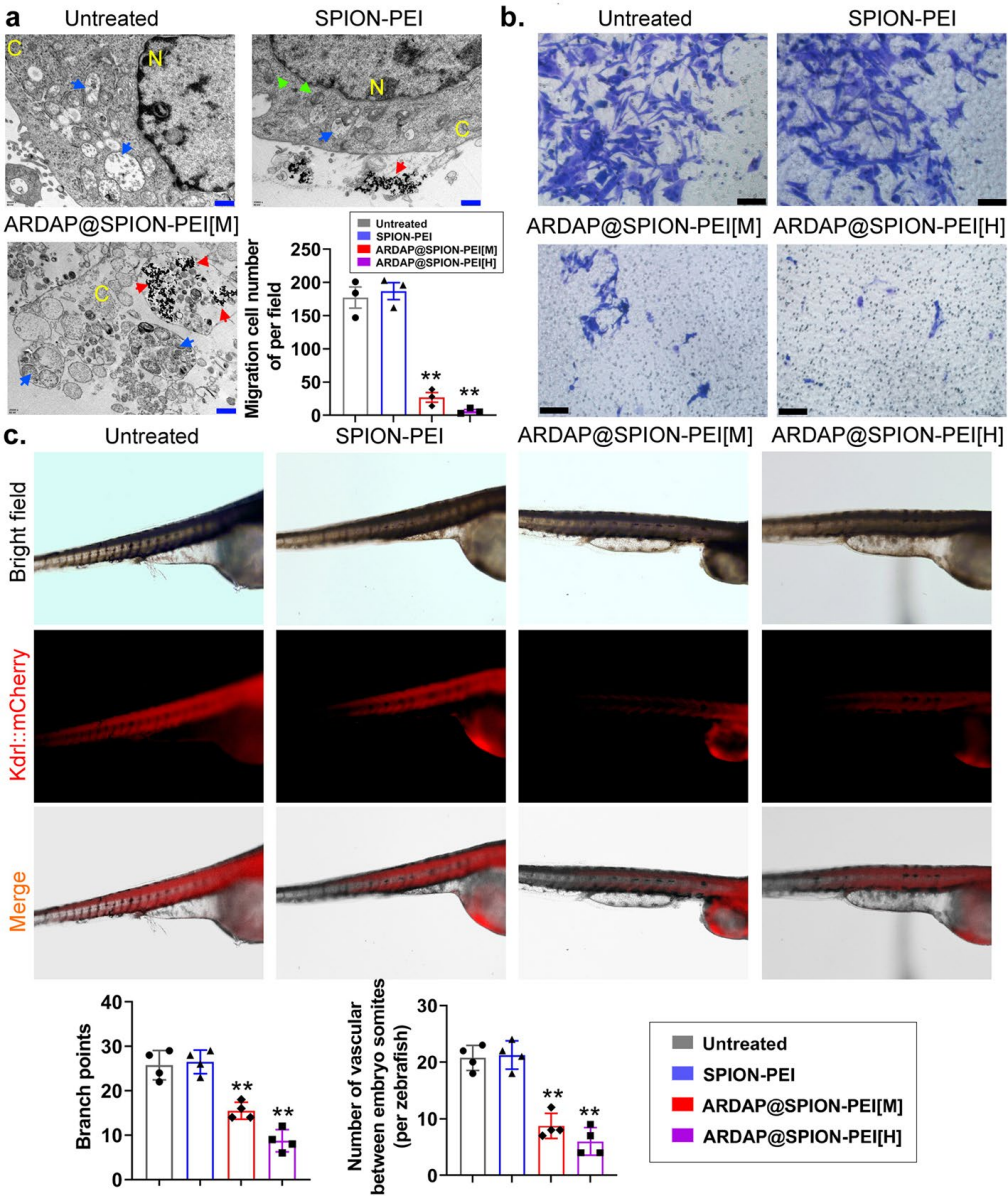
ARDAP@SPION-PEI exposure significantly inhibited in vitro proliferation and migration in mRCSCs. **a** TEM images showing ultrastructural alterations in treated mRCSCs, including organelle degeneration; blue arrows indicate autophagosomes, green arrows indicate normal mitochondria, and red arrows indicate ARDAP@SPION-PEI nanoparticles; C denotes cytoplasm and N denotes nucleus; scale bar = 500 nm. **b** Transwell assay demonstrating significantly reduced migratory capacity following ARDAP@SPION-PEI treatment; scale bar = 20 μm; **P < 0.01 vs. SPION-PEI, Student’s *t* test (*n* = 3). **c** Representative images showing inhibition of ISV and DLAV development in Kdrl::mCherry zebrafish embryos after ARDAP@SPION-PEI treatment; **P < 0.01 vs. SPION-PEI, Student’s *t* test (*n* = 6)

Ultrastructural examination by TEM demonstrated pronounced cellular damage in treated mRCSCs, including cytoplasmic swelling, membrane rupture, nuclear disintegration, and mitochondrial deformation (Fig. 3a). Transwell assays showed significant suppression of migratory capacity in both ARDAP@SPION-PEI[M] and ARDAP@SPION-PEI[H] groups compared with controls (Fig. 3b). In addition, exposure of zebrafish embryos to ARDAP@SPION-PEI[H] for 24 h significantly inhibited ISV and DLAV development relative to the control group (Fig. 3c). These findings demonstrate that ARDAP@SPION-PEI markedly suppresses mRCSC viability, proliferation, migration, and angiogenic potential in vitro.

### ARDAP@SPION-PEI significantly inhibit mRCSC activity in vivo

**Fig. 4:**
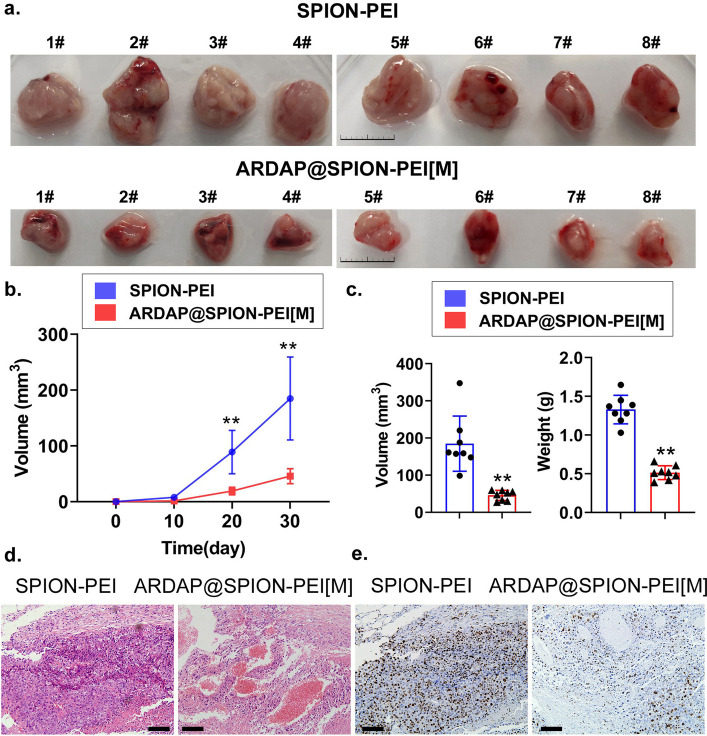
ARDAP@SPION-PEI exposure significantly suppressed in vivo tumor growth derived from mRCSCs. **a** Representative images of subcutaneous tumors in each treatment group (scale bar = 1.0 cm). **b** Tumor growth curves showing significant inhibition in the ARDAP@SPION-PEI group; **P < 0.01 vs. SPION-PEI, Student’s *t* test (*n* = 8). **c** Tumor weight and volume measurements demonstrating significant reduction in treated mice; **P < 0.01 vs. SPION-PEI; *P < 0.05 vs. SPION-PEI, Student’s *t* test (*n* = 8). **d** Hematoxylin and eosin staining showing reduced malignancy and increased necrosis in treated tumors (magnification, 400 × , scale bar = 15 µm). **e** Immunohistochemical staining demonstrating decreased Ki67 expression in tumors from ARDAP@SPION-PEI-treated mice (magnification, 400 × , scale bar = 15 µm)

To evaluate the inhibitory effects of ARDAP@SPION-PEI on mRCSC tumorigenicity in vivo, a subcutaneous tumor-bearing mouse model was established. mRCSCs were inoculated into the dorsal flanks of C57BL/6 mice, followed by intraperitoneal administration of ARDAP@SPION-PEI[M] or SPION-PEI. After 30 days of treatment, the mice were euthanized and tumor tissues were harvested for analysis. Compared with the SPION-PEI control group, the ARDAP@SPION-PEI-treated group exhibited significantly reduced tumor volume and weight (Fig. 4a–c, Fig. S3b, S3c). Histopathological examination using hematoxylin and eosin staining revealed that tumors from the ARDAP@SPION-PEI-treated group displayed fewer atypical nuclei, increased areas of hemorrhage, and extensive necrosis relative to controls (Fig. 4d, Fig. S3a). Immunohistochemical analysis further demonstrated a marked reduction in Ki67 expression, indicating suppressed proliferative activity in tumor tissues following ARDAP@SPION-PEI treatment (Fig. 4e). To assess the impact of ARDAP@SPION-PEI on the tumor immune microenvironment, immunohistochemical staining was performed to detect cytotoxic T cell and macrophage activation markers. The expression levels of CD8 + /IFNγ + and CD8 + /TNFα + cytotoxic T cell markers were significantly increased in tumors from the ARDAP@SPION-PEI-treated group compared with controls. Similarly, type 1 macrophage markers (CD86 + /IFNγ + and CD86 + /TNFα +) were markedly elevated in treated tumors (Fig. S4). These findings indicate that ARDAP@SPION-PEI enhances antitumor immune activation within the tumor microenvironment. To evaluate systemic safety, histological examination of major organs and peripheral blood biochemical analyses were conducted. No significant pathological abnormalities were observed in the heart, lungs, liver, kidneys, or brain across groups, and liver, renal, and cardiac function parameters remained within normal ranges (Fig. S5a, S5b). These results demonstrate that ARDAP@SPION-PEI significantly suppresses mRCSC-driven tumor growth in vivo while exhibiting a favorable safety profile.

### ARDAP@SPION-PEI significantly promote mRCSC PANoptosis

**Fig. 5:**
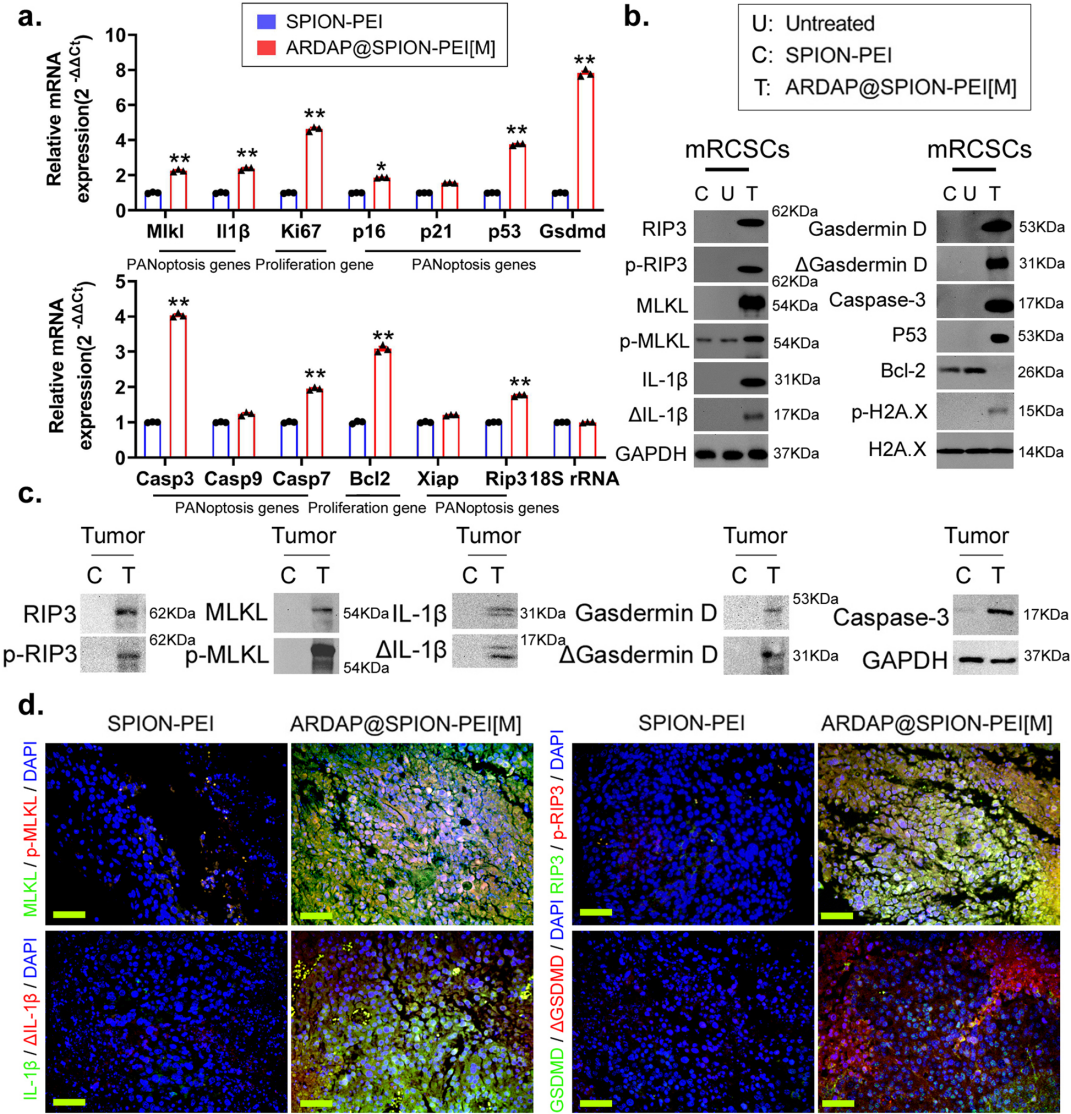
ARDAP@SPION-PEI significantly promoted PANoptosis in mRCSCs in vitro and in vivo. **a** qPCR analysis showing upregulation of PANoptosis-related genes following ARDAP@SPION-PEI treatment. **P < 0.01 vs. SPION-PEI; *P < 0.05 vs. SPION-PEI, Student’s *t* test (*n* = 3). **b** Western blot analysis demonstrating increased expression of PANoptosis-associated proteins in mRCSCs. **c** Western blot analysis of tumor tissues showing elevated PANoptosis markers and phosphorylation levels in the ARDAP@SPION-PEI-treated group. **d** Immunofluorescence staining confirming increased expression of PANoptosis-associated proteins in treated tumors (magnification, 400 × , scale bar = 15 µm)

To determine the mode of cell death induced by ARDAP@SPION-PEI, mRCSCs were treated with specific inhibitors of pyroptosis, apoptosis, necroptosis, or ferroptosis in combination with ARDAP@SPION-PEI[M], and cell viability was assessed using the CCK-8 assay. Co-treatment with any of these inhibitors significantly attenuated the proliferation inhibition induced by ARDAP@SPION-PEI[M] (Fig. S6). These findings suggest that ARDAP@SPION-PEI[M] induces a coordinated cell death program involving multiple pathways, consistent with PANoptosis. Consistently, qPCR analysis demonstrated that ARDAP@SPION-PEI treatment significantly upregulated the mRNA expression of key PANoptosis-related genes, including Rip3, Mlkl, Il1b, Gsdmd, and Casp3, while markedly reducing the expression of the anti-apoptotic factor Bcl2 in vitro (Fig. 5a). Western blot analysis further confirmed increased phosphorylation of RIP3 and MLKL, as well as elevated p-H2A.X levels, accompanied by decreased BCL2 expression in treated mRCSCs (Fig. 5b). In vivo results were consistent with these observations. Tumor tissues from ARDAP@SPION-PEI-treated mice exhibited significantly increased expression of PANoptosis-associated proteins and their phosphorylated forms, together with reduced expression of proliferation-related proteins compared with controls (Fig. 5c). Immunohistochemical analysis showed decreased Ki67 and BCL2 expression in treated tumors, whereas IL-1β, ΔIL-1β, MLKL, p-MLKL, GSDMD, ΔGSDMD, RIP3, p-RIP3, Caspase-3, and Bax were markedly upregulated (Fig. 5d, Fig. S7). These results demonstrate that ARDAP@SPION-PEI robustly induces PANoptosis in mRCSCs both in vitro and in vivo, thereby suppressing tumor cell proliferation.

### The ATAC-seq data were high quality

To characterize the genome-wide chromatin accessibility landscape and elucidate the epigenetic mechanisms underlying ARDAP@SPION-PEI–mediated inhibition of mRCSC activity in vivo, ATAC-seq was performed on samples from each group. Clean reads from all samples were aligned to the mouse reference genome to generate genome-wide accessibility profiles. More than 95% of sequencing reads were successfully mapped to the genome, and over 93% of clean reads were uniquely and properly mapped, indicating high alignment efficiency and data reliability. Library quality was assessed based on fragment size distribution and enrichment patterns of peak signals (Fig. S8a and S8b; Supplementary Data ATAC-seq all peaks data) [31, 32]. The fragment length distribution displayed the expected nucleosome periodicity, confirming appropriate chromatin fragmentation. Genome-wide peak annotation showed that the majority of sequencing signals were enriched within ±2 kb of transcription start sites (TSS), consistent with active regulatory regions and transcriptional control elements (Fig. S8c). Pearson correlation analysis of read densities demonstrated strong reproducibility within groups and clear distinctions between the SPION-PEI control and ARDAP@SPION-PEI treatment groups. Correlation coefficients between biological replicates approached 1.0, further confirming high data consistency and quality (Fig. S8d). Additionally, chromosome-wide distribution maps revealed consistent sequencing signal intensity and peak enrichment patterns across samples (Fig. S8e). The read distribution, peak enrichment profiles, and reproducibility analyses confirm that the ATAC-seq data are of high quality and suitable for downstream chromatin accessibility and mechanistic analyses.

### ARDAP@SPION-PEI exert pharmacological effects in mRCSCs through chromatin accessibility alterations

**Fig. 6:**
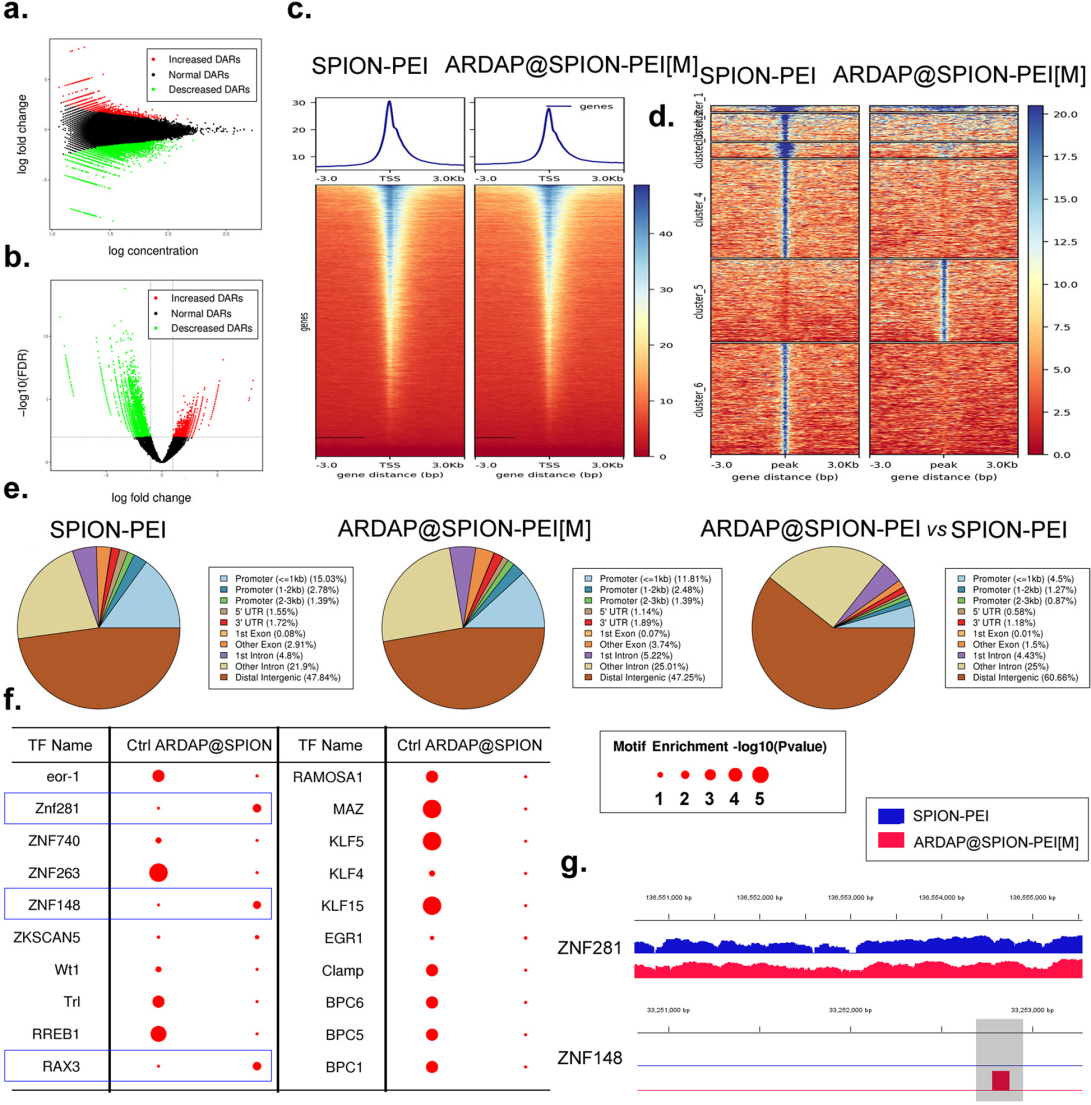
ARDAP@SPION-PEI significantly altered chromatin accessibility in mRCSCs. **a** MA plot showing the distribution of differential chromatin accessibility peaks between groups. **b** Volcano plot illustrating significantly altered peaks. **c** Heatmap showing density distribution of ATAC-seq reads within ±3 kb of transcription start sites. **d** Cluster analysis and differential enrichment heatmap of ATAC-seq peaks across samples. **e** Genomic annotation of differential peak regions relative to gene functional elements. **f** Motif enrichment analysis identifying transcription factor binding motifs enriched in each group. **g** Integrated Genomics Viewer visualization of ATAC-seq read enrichment across representative genomic regions

**Fig. 7:**
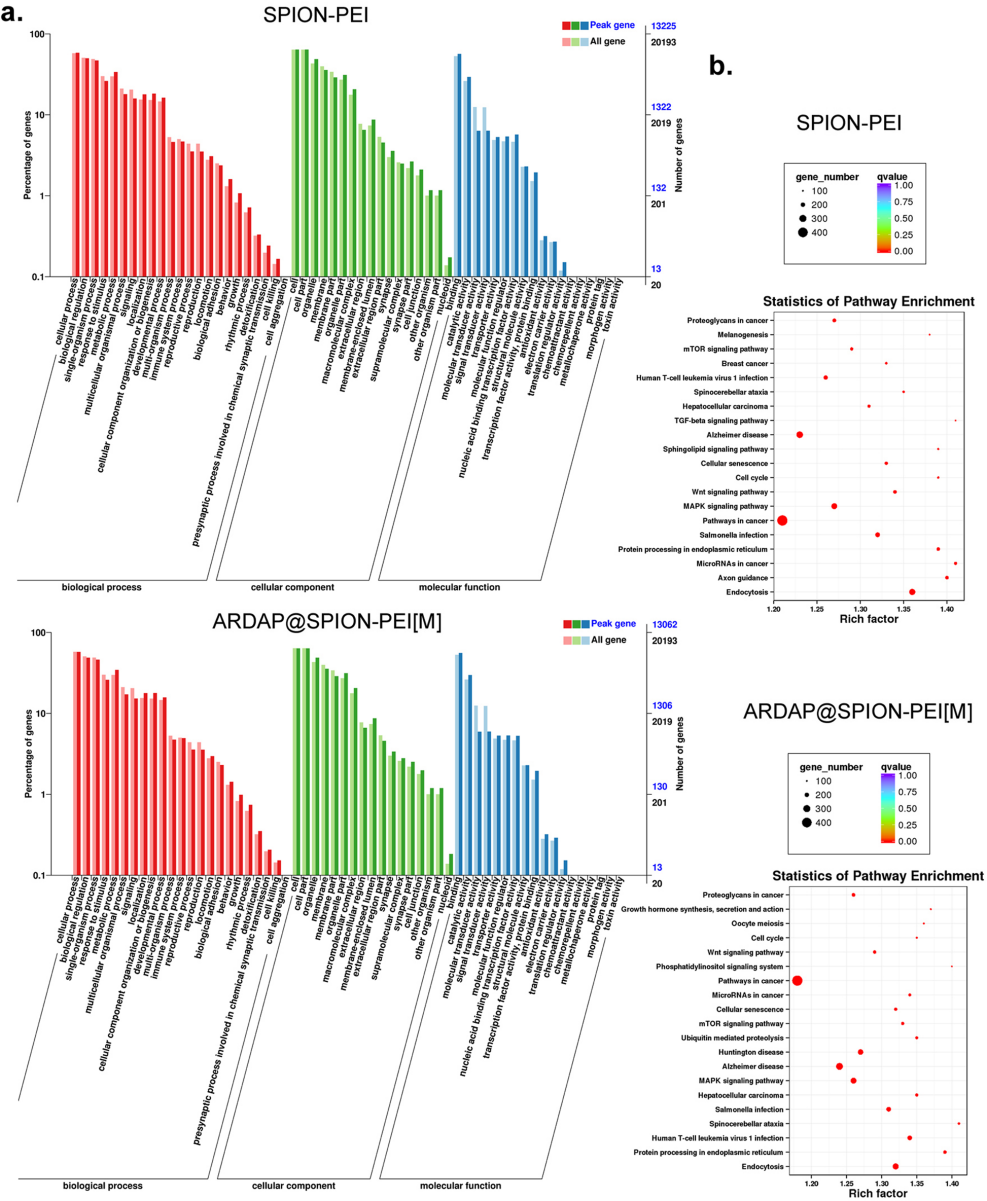
Functional annotation of differential chromatin accessibility peaks identified by ATAC-seq. **a** Gene Ontology enrichment analysis of promoter-associated peak genes. **b** KEGG pathway enrichment analysis of promoter-associated peak genes

To investigate global changes in chromatin accessibility induced by ARDAP@SPION-PEI, we performed correlation analyses of peak accessibility intensity and fold changes using MA and volcano plots. Genome-wide chromatin accessibility maps revealed substantial differences in open chromatin regions between the SPION-PEI control and ARDAP@SPION-PEI treatment groups (Fig. 6a, b). Using fold change ≥ 2 and P ≤ 0.01 as criteria for differential accessibility, we identified 168,566 and 196,339 peaks specific to the SPION-PEI control and ARDAP@SPION-PEI treatment groups, respectively (Fig. 6a, b). Among these peaks, 2,639 showed significant increases, 6,618 showed significant decreases, and 7,793 exhibited no significant differences between groups (Fig. 6a, b). As expected, ATAC-seq signals were enriched within open chromatin regions and were positively associated with transcriptional activity. Heatmap analysis demonstrated pronounced enrichment of sequencing reads within ±3 kb of transcription start sites (TSS), as well as at transcription termination sites (Fig. 6c). Clustering analysis categorized differentially accessible peaks into six distinct groups with unique accessibility patterns (Fig. 6d). Annotation of peak regions revealed that the majority were located in promoter regions, 5′ untranslated regions (UTRs), first exon regions, and intergenic regions (Fig. 6e), consistent with regulatory elements controlling gene transcription. Motif enrichment analysis identified differential activation of transcription factors between groups. ZNF263, RREB1, MAZ, KLF5, and KLF15 were enriched in the SPION-PEI control group, whereas ZNF281, ZNF148, and RAX3 were prominently enriched in the ARDAP@SPION-PEI treatment group (Fig. 6f). Visualization of ATAC-seq signal tracks further demonstrated a marked increase in chromatin accessibility at the ZNF148 locus in treated samples (Fig. 6g). Gene Ontology analysis showed enrichment of genes associated with biological processes, cellular components, and molecular functions within accessible chromatin regions in both groups (Fig. 7a). Kyoto Encyclopedia of Genes and Genomes (KEGG) pathway analysis revealed significant enrichment of signal transduction pathways, including cancer-related pathways, endocytosis, and MAPK signaling (Fig. 7b). These findings indicate that ARDAP@SPION-PEI exerts its pharmacological effects in mRCSCs through widespread chromatin remodeling, with prominent activation of ZNF148-associated regulatory networks.

### ARDAP@SPION-PEI-mediated ZNF148 activity promotes transcriptional activation of PANoptosis key genes in mRCSCs

**Fig. 8:**
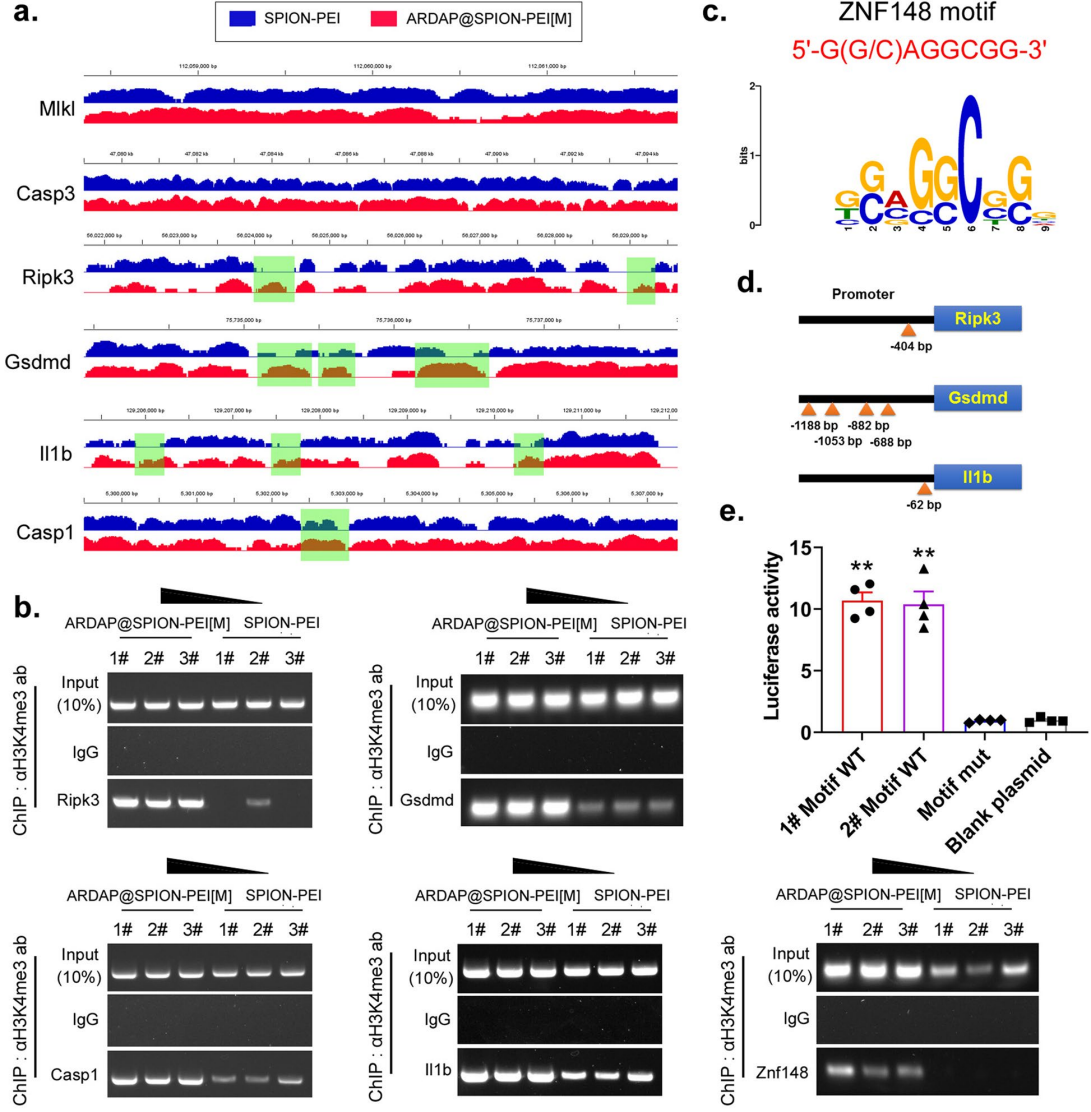
ARDAP@SPION-PEI-mediated ZNF148 activity promoted transcriptional activation of PANoptosis key genes in mRCSCs. **a** Integrated Genomics Viewer visualization of ATAC-seq peaks at PANoptosis-related gene loci. **b** ChIP-PCR analysis demonstrating increased enrichment of H3K4me3 at promoters of PANoptosis genes following ARDAP@SPION-PEI treatment (*n* = 3). **c** Bioinformatic identification of the ZNF148 DNA-binding motif (5′-G(G/C)AGGCGG-3′). **d** Motif analysis showing multiple ZNF148-binding sites within the Ripk3, Gsdmd, and Il1b promoters. **e** Luciferase reporter assay demonstrating significantly increased luciferase activity when the wild-type ZNF148-binding motif was present in the promoter construct. **P < 0.01 vs. blank plasmid DNA, Student’s *t* test (*n* = 3)

Our qPCR and Western blot analyses demonstrated that ARDAP@SPION-PEI treatment significantly upregulated key PANoptosis-related factors in mRCSCs. Consistent with these findings, visualization of ATAC-seq data revealed pronounced chromatin accessibility peaks at the loci of *Ripk3*, *Gsdmd*, *Il1b*, and *Casp1* in the ARDAP@SPION-PEI treatment group compared with the SPION-PEI control group (Fig. 8a), indicating enhanced transcriptional accessibility at these genomic regions. To further elucidate the mechanism by which ARDAP@SPION-PEI regulates PANoptosis gene expression, we performed ChIP-PCR and luciferase reporter assays. ChIP-PCR analysis demonstrated significantly increased enrichment of H3K4me3, a marker of transcriptionally active chromatin, at the promoter regions of *Ripk3*, *Gsdmd*, *Il1b*, *Casp1*, and *Znf148* in the ARDAP@SPION-PEI-treated group relative to controls (Fig. 8b), suggesting enhanced promoter activation. Bioinformatic motif analysis identified multiple Znf148-binding motifs (5′-G(G/C)AGGCGG-3′) within the promoter regions of *Ripk3*, *Gsdmd*, and *Il1b* (Fig. 8c, d). Luciferase reporter assays further confirmed that insertion of wild-type Znf148-binding motifs into promoter constructs significantly increased luciferase activity, demonstrating direct transcriptional activation mediated by ZNF148 (Fig. 8e). These results indicate that ARDAP@SPION-PEI enhances chromatin accessibility and promotes H3K4me3 enrichment at PANoptosis gene promoters, thereby facilitating ZNF148-dependent transcriptional activation of key PANoptosis genes in mRCSCs.

### Silencing *ZNF148* attenuates ARDAP@SPION-PEI-mediated activation of PANoptosis key genes in mRCSCs

To determine whether ZNF148 is required for ARDAP@SPION-PEI–induced activation of PANoptosis genes, endogenous *ZNF148* expression was silenced in mRCSCs using siRNA. The expression levels of PANoptosis-related genes and cell proliferation were subsequently evaluated by qPCR, Western blotting, and CCK-8 assays. CCK-8 analysis showed that treatment with ARDAP@SPION-PEI combined with siRNA-Mock significantly increased the proliferation inhibition rate compared with the Untreated group. In contrast, co-treatment with ARDAP@SPION-PEI and siRNA-ZNF148 markedly reduced the proliferation inhibition rate relative to the ARDAP@SPION-PEI + siRNA-Mock group (Fig. S9a), indicating that *ZNF148* knockdown attenuated ARDAP@SPION-PEI–induced cytotoxicity. Consistently, qPCR analysis demonstrated that ARDAP@SPION-PEI + siRNA-Mock treatment significantly upregulated *Znf148* and PANoptosis-associated genes, including *Rip3*, *Mlkl*, *Il1b*, *Gsdmd*, and *Casp3*, while reducing the expression of proliferation-related genes *Bcl2* and *Ki67* compared with the Untreated group. However, *Znf148* knockdown markedly diminished the induction of these PANoptosis-related genes in the ARDAP@SPION-PEI + siRNA-ZNF148 group relative to the ARDAP@SPION-PEI + siRNA-Mock group (Fig. S9b). Western blot analysis further confirmed that the protein levels of PANoptosis-associated factors were significantly reduced following *ZNF148* silencing compared with ARDAP@SPION-PEI + siRNA-Mock treatment (Fig. S9c). These findings demonstrate that ZNF148 is essential for ARDAP@SPION-PEI–mediated transcriptional activation of PANoptosis genes in mRCSCs (Fig. S10).

## Discussion

CSCs are widely recognized as key drivers of tumor metastasis, recurrence, therapeutic resistance, and poor clinical outcomes [1, 3–5, 7, 37]. Current cancer treatment strategies primarily rely on surgical resection, radiotherapy, and chemotherapy [1, 3–5, 7, 37]. However, due to their intrinsic resistance to conventional chemotherapeutic agents and strong proliferative capacity, CSCs are often insufficiently eliminated by standard therapies. Therefore, the development of therapeutic agents specifically targeting CSCs remains an urgent clinical priority. ARDAP and its derivatives were originally developed by Silvestri et al. as a class of small-molecule compounds with potent anticancer activity [13, 14]. By binding to the colchicine site of tubulin, ARDAP disrupts microtubule assembly and polymerization, thereby inhibiting cancer cell growth [13, 14]. Nevertheless, whether ARDAP exerts similar cytotoxic effects on CSCs has remained unclear. Subsequent structural optimization, including replacement of the 4-aminophenyl group on the pyrrole nucleus with a five- or six-membered heterocyclic ring while retaining key methyl and fluorine substituents on the 1-phenyl ring, further enhanced the biological activity of the derivative RS-5645 [13, 14].

SPIONs have emerged as versatile nanocarrier platforms with several distinct advantages [15–17]. Under an external magnetic field, they can facilitate the delivery of nucleic acids and small-molecule compounds into mammalian cells, overcome intracellular and extracellular barriers via magnetic guidance, and increase local drug concentrations to enhance therapeutic efficacy. Surface functionalization with cationic polymers, such as polyethyleneimine, dendrimers, glucose, chitosan, or cationic liposomes, further improves loading capacity and delivery efficiency. Owing to their structural properties, SPIONs can encapsulate high drug concentrations and enable rapid intracellular accumulation, thereby enhancing cytotoxic effects. Previous studies have demonstrated that SPION-based delivery systems carrying noncoding RNAs effectively suppress the growth of glioma stem cells and ovarian CSCs both in vitro and in vivo [19, 21–23, 25]. Building upon these advantages, we constructed ARDAP@SPION-PEI and evaluated its effects on CSCs. Our findings demonstrate that ARDAP@SPION-PEI markedly inhibits mRCSC proliferation and migration in vitro, reduces tumorigenicity in vivo, and suppresses angiogenesis in zebrafish models. These results indicate that ARDAP@SPION-PEI exerts multifaceted antitumor activity against mRCSCs and highlight its potential as a nanotherapeutic strategy for targeting CSCs.

Previous studies have demonstrated that ARDAP induces cancer cell death through multiple mechanisms, depending on tumor type [10–14]. For example, ARDAP has been shown to trigger ferroptosis in glioma and ovarian cancer cells by modulating the expression of genes such as *PTGS2*, *NFE2L2*, *SAT1*, *AKR1C1*, and *GPX4* [11]. In addition, a structurally optimized ARDAP derivative promotes apoptosis in leukemia, bladder cancer, and ovarian cancer cells by suppressing BCL2 expression and enhancing CASP3 activation [10, 13]. These findings suggest that ARDAP-based compounds may exert antitumor effects through diverse and context-dependent cell death pathways. In the present study, ARDAP@SPION-PEI inhibited cell-cycle progression and increased *Casp1* expression in mRCSCs, indicating activation of an inflammatory cell death program. Given the concurrent induction of apoptosis- and necroptosis-related markers, these results support the involvement of PANoptosis.

PANoptosis is an inflammatory programmed cell death pathway regulated by the PANoptosome complex and integrates the molecular features of pyroptosis, apoptosis, and necroptosis [26–30]. Activation of PANoptosis is mediated by upstream sensors and signaling cascades that assemble into PANoptosomes, which function as molecular scaffolds and signaling hubs coordinating downstream effector activation [26–30]. The PANoptosome typically consists of three functional components: pathogen- and damage-associated molecular pattern sensors, including ZBP1 and NLRP3; adaptor proteins such as ASC and FADD; and catalytic effectors including RIPK1, RIPK3, CASP1, and CASP8 [26–30]. Distinct upstream molecules, including ZBP1, RIPK1, and AIM2, can sense specific stimuli and initiate the formation of corresponding PANoptosome complexes [38, 39]. Conversely, regulatory molecules such as TAK1, PSTPIP2, SHARPIN, HOIP, HOIL1, and A20 suppress PANoptosome activation [26–30]. Although initially characterized in infectious and inflammatory diseases, accumulating evidence suggests that PANoptosis may also represent a promising therapeutic mechanism in cancer [26–30].

Consistent with this concept, our qPCR, Western blotting, and immunohistochemical analyses demonstrated that ARDAP@SPION-PEI markedly increased the expression of PANoptosis-related markers in mRCSCs compared with controls, supporting the induction of PANoptosis. Importantly, ATAC-seq and luciferase reporter assays further revealed that activation of the PANoptosis pathway was associated with enhanced chromatin accessibility and ZNF148-dependent transcriptional activation. Together, these findings indicate that ARDAP@SPION-PEI induces PANoptosis in mRCSCs through coordinated epigenetic remodeling and transcriptional regulation.

Our study demonstrates that ARDAP@SPION-PEI alters chromatin accessibility in mRCSCs, thereby influencing the transcriptional activity and expression of target genes. However, chromatin accessibility is regulated by multiple epigenetic mechanisms, including covalent histone modifications and DNA methylation. Based on our findings, it can be inferred that ARDAP@SPION-PEI exerts its therapeutic effects, at least in part, through modulation of epigenetic regulation in mRCSCs. Because both histone modifications and DNA methylation are enzymatically controlled processes, it is plausible that ARDAP@SPION-PEI may directly or indirectly regulate the expression or activity of specific epigenetic enzymes, thereby driving changes in chromatin accessibility. Nevertheless, as this study primarily focused on pharmacodynamic characterization, we did not comprehensively investigate the interaction between ARDAP@SPION-PEI and epigenetic modifying enzymes. Elucidating these molecular interactions will be an important direction for future research.

In addition, translational considerations remain to be addressed. Future studies will focus on improving tumor specificity and selective targeting of CSCs, potentially through ligand conjugation strategies to enhance active targeting efficiency. Although ARDAP@SPION-PEI induces tumor suppression via PANoptosis, this form of inflammatory cell death may also provoke excessive inflammatory responses that could damage surrounding normal tissues. Therefore, strategies to facilitate timely clearance of residual nanocomposites, such as enhancing phagocytic cell activation, should be explored to minimize potential off-target effects. Finally, optimization of scalable and reproducible synthesis methods for the nanocarrier system will be necessary to support future clinical translation.

In summary, we developed ARDAP@SPION-PEI and systematically evaluated its antitumor efficacy and underlying epigenetic mechanisms in mRCSCs both in vitro and in vivo. Our findings demonstrate that ARDAP@SPION-PEI remodels chromatin accessibility, leading to enhanced *ZNF148* expression and promoter activation of key PANoptosis-related genes. This epigenetic reprogramming ultimately drives PANoptosis and suppresses tumor stem cell proliferation and tumorigenicity. These results establish a mechanistically defined nanotherapeutic strategy that targets CSCs through coordinated chromatin remodeling and inflammatory programmed cell death activation.

## Materials and methods

A detailed description of all experimental procedures is provided in the Supplementary Data Materials and Methods.

### ARDAP@SPION-PEI preparation

SPION-PEI (100 mg; Novobio Co., Ltd., Shanghai, China) were mixed with 100 mg of ARDAP in 1.0 mL of phosphate buffer (0.2 mol/L Na_2_HPO_4_, pH 6.0) and subjected to ultrasonication for 60 min at room temperature to facilitate drug adsorption. The resulting ARDAP@SPION-PEI nanocomposites were isolated using a neodymium magnet and washed three times with distilled water and absolute ethanol to remove unbound ARDAP. The purified nanocomposites were subsequently vacuum-dried at room temperature and stored at 4 °C until further use.

### mRCSC flow cytometry sorting, enrichment, and intervention

As previously described [9, 40], RENCA cells (murine renal carcinoma cell line) were resuspended at a density of 2 × 10^8^ cells/mL in 0.5 mL of ice-cold sterile phosphate-buffered saline (PBS; HyClone Laboratories, Logan, UT, USA). Cells were incubated with 5 μL anti-mouse CD105-FITC antibody (Miltenyi Biotec, Bergisch Gladbach, Germany) and 5 μL anti-mouse CD44-PE antibody (Miltenyi Biotec) to achieve a final antibody concentration of 0.01 mg/mL. The mixture was incubated at 4 °C for 30 min in the dark. After incubation, cells were washed twice with ice-cold PBS and subjected to fluorescence-activated cell sorting using a BD FACSAria III flow cytometer (BD Biosciences, San Jose, CA, USA) to isolate and enrich CD44 + /CD105 + mRCSCs. Sorted cells were adjusted to a density of 1,000 cells/mL and cultured under sphere-forming conditions in DMEM/F12 medium (HyClone) supplemented with 10 ng/mL basic fibroblast growth factor, 10 ng/mL epidermal growth factor, 5 μg/mL insulin, and 0.5% bovine serum albumin (Sigma-Aldrich, St. Louis, MO, USA). Cells were expanded to the third passage before subsequent experiments. For treatment studies, cells were divided into medium-dose, high-dose, and control groups and exposed to ARDAP@SPION-PEI[M], ARDAP@SPION-PEI[H] (80 nM), or SPION-PEI at equivalent concentrations, respectively. A blank control group received no treatment. All groups were incubated with the respective agents for 24 h prior to analysis.

### In vivo xenograft experiments

Log-phase mRCSCs (1 × 10^6^ cells per mouse) were suspended in sterile PBS and subcutaneously injected into the dorsal flanks of male BALB/c mice (6–8 weeks old), obtained from the Experimental Animal Center of Shanghai University of Traditional Chinese Medicine (Shanghai, China). Tumor growth was monitored regularly, and when tumors reached approximately 2 mm in diameter, mice were randomly assigned to two groups (n = 8 per group). Mice received intraperitoneal injections of 100 μL ARDAP@SPION-PEI[M] (25 mg/kg) or SPION-PEI at an equivalent dose once every two days, as previously described [10, 11]. Treatment was continued for 30 consecutive days. At the end of the intervention period, mice were euthanized, and tumors were excised and weighed. Tumor volume (mm^3^) was calculated using the formula (a × b^2^)/2, where a represents the longest diameter and b the shortest diameter (both in mm). All animal procedures were conducted in accordance with the National Institutes of Health guidelines for the care and use of laboratory animals.

### ATAC-seq

ATAC-seq, including sample preparation and high-throughput sequencing, was performed according to the standard protocol recommended by Illumina [36] and conducted by Biomarker Technologies Co., Ltd. (Beijing, China). Briefly, single-cell suspensions were prepared from each experimental group, and nuclei were isolated under optimized lysis conditions. The Tn5 transposase-mediated tagmentation reaction was subsequently carried out to simultaneously fragment accessible chromatin regions and insert sequencing adapters. Following purification, library amplification was performed by PCR, and the amplified products were subjected to high-throughput sequencing. Raw sequencing reads were processed to remove adapter sequences and low-quality reads, generating clean reads for downstream analysis. Clean reads were aligned to the mouse reference genome to determine genomic localization. Peak calling was performed based on read enrichment across the genome, and identified peaks were annotated to genomic features, including promoter, exon, intron, and intergenic regions. Differentially accessible peaks were further analyzed to identify genes associated with altered chromatin accessibility. Motif enrichment analysis was subsequently conducted on differential peak regions to predict potential transcription factor binding sites.

### Statistical analysis

All data are presented as Mean ± SD. The comparison of means between the two groups was performed using Student's *t*-test. The experiment was repeated three times, with *P* < 0.05 indicating statistical significance.

## Supplementary Information

Supplementary Material 1.

Supplementary Material 2.

## Data Availability

The raw sequence data reported in this paper have been deposited in the Genome Sequence Archive (Genomics, Proteomics & Bioinformatics 2025) in National Genomics Data Center (Nucleic Acids Res 2025), China National Center for Bioinformation/Beijing Institute of Genomics, Chinese Academy of Sciences (GSA number: CRA042443) that are publicly accessible at https://ngdc.cncb.ac.cn/gsa-human. The data that supports the findings of this study are available in the supplementary material of this article.

## References

[1] Jonasch E, Gao J, Rathmell WK. Renal cell carcinoma. BMJ. 2014;349:g4797. 10.1136/bmj.g4797. 25385470 10.1136/bmj.g4797 PMC4707715

[2] Santini D, Tonini G. Treatment of advanced renal-cell carcinoma. N Engl J Med. 2016;374(9):888–9. 10.1056/NEJMc1515613. 26962913 10.1056/NEJMc1515613

[3] Capitanio U, Montorsi F. Renal cancer. Lancet. 2016;387(10021):894–906. 10.1016/S0140-6736(15)00046-X. 26318520 10.1016/S0140-6736(15)00046-X

[4] Jordan CT, Guzman ML, Noble M. Cancer stem cells. N Engl J Med. 2006;355(12):1253–61. 10.1056/NEJMra061808. 16990388 10.1056/NEJMra061808

[5] Filipovic A, Stebbing J, Giamas G. Cancer stem cells--therapeutic targeting or therapy? Lancet Oncol. 2013;14(7):579–80. 10.1016/S1470-2045(13)70258-4. 23725700 10.1016/S1470-2045(13)70258-4

[6] Tomasetti C, Durrett R, Kimmel M, Lambert A, Parmigiani G, Zauber A, et al. Role of stem-cell divisions in cancer risk. Nature. 2017;548(7666):E13–4. 10.1038/nature23302. 28796214 10.1038/nature23302

[7] Greten FR. Cancer: tumour stem-cell surprises. Nature. 2017;543(7647):626–7. 10.1038/543626a. 28358084 10.1038/543626a

[8] Shin SW. Clinical and therapeutic implications of cancer stem cells. N Engl J Med. 2019;381(10):e19. 10.1056/NEJMc1908886. 31483981 10.1056/NEJMc1908886

[9] Si Y, Liu J, Shen H, Zhang C, Wu Y, Huang Y, et al. Fisetin decreases TET1 activity and CCNY/CDK16 promoter 5hmC levels to inhibit the proliferation and invasion of renal cancer stem cell. J Cell Mol Med. 2019;23(2):1095–105. 10.1111/jcmm.14010. 30411496 10.1111/jcmm.14010 PMC6349178

[10] La Regina G, Bai R, Coluccia A, Famiglini V, Passacantilli S, Naccarato V, et al. 3-Aroyl-1,4-diarylpyrroles inhibit chronic myeloid leukemia cell growth through an interaction with tubulin. ACS Med Chem Lett. 2017;8(5):521–6. 10.1021/acsmedchemlett.7b00022. 28523104 10.1021/acsmedchemlett.7b00022 PMC5430391

[11] Puxeddu M, Shen H, Bai R, Coluccia A, Nalli M, Mazzoccoli C, et al. Structure-activity relationship studies and in vitro and in vivo anticancer activity of novel 3-aroyl-1,4-diarylpyrroles against solid tumors and hematological malignancies. Eur J Med Chem. 2020;185:111828. 10.1016/j.ejmech.2019.111828. 31727471 10.1016/j.ejmech.2019.111828

[12] Liu T, Wu J, Han C, Gong Z, Regina G, Chen J, et al. RS-5645 attenuates inflammatory cytokine storm induced by SARS-CoV-2 spike protein and LPS by modulating pulmonary microbiota. Int J Biol Sci. 2021;17(13):3305–19. 10.7150/ijbs.63329. 34512148 10.7150/ijbs.63329 PMC8416739

[13] Nalli M, Di Magno L, Wen Y, Liu X, D’Ambrosio M, Puxeddu M, et al. Novel N-(Heterocyclylphenyl)benzensulfonamide Sharing an Unreported Binding Site with T-Cell Factor 4 at the beta-Catenin Armadillo Repeats Domain as an Anticancer Agent. ACS Pharmacol Transl Sci. 2023;6(7):1087–103. 10.1021/acsptsci.3c00092. 37470018 10.1021/acsptsci.3c00092 PMC10353061

[14] Mazzoccoli C, Crispo F, Laurenzana I, Pietrafesa M, Sisinni L, Lerose R, et al. Biological evaluation of [4-(4-aminophenyl)-1-(4-fluorophenyl)-1H-pyrrol-3-yl](3,4,5-trimethoxyphenyl)methanone as potential antineoplastic agent in 2D and 3D breast cancer models. Arch Pharm (Weinheim). 2023;356(10):e2300354. 10.1002/ardp.202300354. 37603378 10.1002/ardp.202300354

[15] Li L, Jiang W, Luo K, Song H, Lan F, Wu Y, et al. Superparamagnetic iron oxide nanoparticles as MRI contrast agents for non-invasive stem cell labeling and tracking. Theranostics. 2013;3(8):595–615. 10.7150/thno.5366. 23946825 10.7150/thno.5366 PMC3741608

[16] Vangijzegem T, Lecomte V, Ternad I, Van Leuven L, Muller RN, Stanicki D et al. Superparamagnetic Iron Oxide Nanoparticles (SPION): From Fundamentals to State-of-the-Art Innovative Applications for Cancer Therapy. Pharmaceutics. 2023;15(1). 10.3390/pharmaceutics15010236. 10.3390/pharmaceutics15010236 PMC9861355 36678868

[17] Rezaei B, Tay ZW, Mostufa S, Manzari ON, Azizi E, Ciannella S, et al. Magnetic nanoparticles for magnetic particle imaging (MPI): design and applications. Nanoscale. 2024. 10.1039/d4nr01195c. 38809214 10.1039/d4nr01195c

[18] Huang Y, Du X, Liu T, Liu Q. siRNA@superparamagnetic iron oxide nanoparticles attenuate physiological toxicity of DEHP by suppressing autophagy pathway activities in Caenorhabditis elegans. Ecotoxicol Environ Saf. 2022;229:113083. 10.1016/j.ecoenv.2021.113083. 34915219 10.1016/j.ecoenv.2021.113083

[19] Pan Z, Shi Z, Wei H, Sun F, Song J, Huang Y, et al. Magnetofection based on superparamagnetic iron oxide nanoparticles weakens glioma stem cell proliferation and invasion by mediating high expression of MicroRNA-374a. J Cancer. 2016;7(11):1487–96. 10.7150/jca.15515. 27471565 10.7150/jca.15515 PMC4964133

[20] Fang K, Liu P, Dong S, Guo Y, Cui X, Zhu X, et al. Magnetofection based on superparamagnetic iron oxide nanoparticle-mediated low lncRNA HOTAIR expression decreases the proliferation and invasion of glioma stem cells. Int J Oncol. 2016;49(2):509–18. 10.3892/ijo.2016.3571. 27277755 10.3892/ijo.2016.3571 PMC4922836

[21] Liu T, Zhang H, Zheng J, Lin J, Huang Y, Chen J, et al. SPION-mediated miR-141 promotes the differentiation of HuAESCs into dopaminergic neuron-like cells via suppressing lncRNA-HOTAIR. J Cell Mol Med. 2018;22(4):2299–310. 10.1111/jcmm.13512. 29411538 10.1111/jcmm.13512 PMC5867164

[22] Gao Y, Qian H, Tang X, Du X, Wang G, Zhang H, et al. Superparamagnetic iron oxide nanoparticle-mediated expression of miR-326 inhibits human endometrial carcinoma stem cell growth. Int J Nanomedicine. 2019;14:2719–31. 10.2147/IJN.S200480. 31114192 10.2147/IJN.S200480 PMC6497851

[23] Pan Z, Huang Y, Qian H, Du X, Qin W, Liu T. Superparamagnetic iron oxide nanoparticles drive miR-485-5p inhibition in glioma stem cells by silencing Tie1 expression. Int J Biol Sci. 2020;16(7):1274–87. 10.7150/ijbs.42887. 32174801 10.7150/ijbs.42887 PMC7053326

[24] Ni Z, Nie X, Zhang H, Wang L, Geng Z, Du X, et al. Atranorin driven by nano materials SPION lead to ferroptosis of gastric cancer stem cells by weakening the mRNA 5-hydroxymethylcytidine modification of the Xc-/GPX4 axis and its expression. Int J Med Sci. 2022;19(11):1680–94. 10.7150/ijms.73701. 36237989 10.7150/ijms.73701 PMC9553860

[25] Huang Y, Lin J, Xiong Y, Chen J, Du X, Liu Q, et al. Superparamagnetic iron oxide nanoparticles induce ferroptosis of human ovarian cancer stem cells by weakening cellular autophagy. J Biomed Nanotechnol. 2020;16(11):1612–22. 10.1166/jbn.2020.2991. 33461653 10.1166/jbn.2020.2991

[26] Wang Y, Kanneganti TD. From pyroptosis, apoptosis and necroptosis to PANoptosis: a mechanistic compendium of programmed cell death pathways. Comput Struct Biotechnol J. 2021;19:4641–57. 10.1016/j.csbj.2021.07.038. 34504660 10.1016/j.csbj.2021.07.038 PMC8405902

[27] Christgen S, Zheng M, Kesavardhana S, Karki R, Malireddi RKS, Banoth B, et al. Identification of the PANoptosome: a molecular platform triggering pyroptosis, apoptosis, and necroptosis (PANoptosis). Front Cell Infect Microbiol. 2020;10:237. 10.3389/fcimb.2020.00237. 32547960 10.3389/fcimb.2020.00237 PMC7274033

[28] Samir P, Malireddi RKS, Kanneganti TD. The PANoptosome: a deadly protein complex driving pyroptosis, apoptosis, and necroptosis (PANoptosis). Front Cell Infect Microbiol. 2020;10:238. 10.3389/fcimb.2020.00238. 32582562 10.3389/fcimb.2020.00238 PMC7283380

[29] Sun X, Yang Y, Meng X, Li J, Liu X, Liu H. PANoptosis: Mechanisms, biology, and role in disease. Immunol Rev. 2024;321(1):246–62. 10.1111/imr.13279. 37823450 10.1111/imr.13279

[30] Malireddi RKS, Kesavardhana S, Kanneganti TD. ZBP1 and TAK1: master regulators of NLRP3 inflammasome/pyroptosis, apoptosis, and necroptosis (PAN-optosis). Front Cell Infect Microbiol. 2019;9:406. 10.3389/fcimb.2019.00406. 31850239 10.3389/fcimb.2019.00406 PMC6902032

[31] Members C-N, Partners. Database resources of the National Genomics Data Center, China National Center for Bioinformation in 2025. Nucleic Acids Res. 2025;53(D1):D30–44. 10.1093/nar/gkae978. 39530327 10.1093/nar/gkae978 PMC11701749

[32] Zhang S, Chen X, Jin E, Wang A, Chen T, Zhang X et al. The GSA Family in 2025: A Broadened Sharing Platform for Multi-omics and Multimodal Data. Genomics Proteomics Bioinformatics. 2025;23(4). 10.1093/gpbjnl/qzaf072. 10.1093/gpbjnl/qzaf072 PMC12451262 40857552

[33] Lin JF, Wang TT, Huang RZ, Tan YT, Chen DL, Ju HQ. PANoptosis in cancer: bridging molecular mechanisms to therapeutic innovations. Cell Mol Immunol. 2025;22(9):996–1011. 10.1038/s41423-025-01329-z. 40721869 10.1038/s41423-025-01329-z PMC12398562

[34] He P, Ma Y, Wu Y, Zhou Q, Du H. Exploring PANoptosis in breast cancer based on scRNA-seq and bulk-seq. Front Endocrinol (Lausanne). 2023;14:1164930. 10.3389/fendo.2023.1164930. 37455906 10.3389/fendo.2023.1164930 PMC10338225

[35] Gao J, Xiong A, Liu J, Li X, Wang J, Zhang L, et al. PANoptosis: bridging apoptosis, pyroptosis, and necroptosis in cancer progression and treatment. Cancer Gene Ther. 2024;31(7):970–83. 10.1038/s41417-024-00765-9. 38553639 10.1038/s41417-024-00765-9 PMC11257964

[36] Grandi FC, Modi H, Kampman L, Corces MR. Chromatin accessibility profiling by ATAC-seq. Nat Protoc. 2022;17(6):1518–52. 10.1038/s41596-022-00692-9. 35478247 10.1038/s41596-022-00692-9 PMC9189070

[37] De Meerleer G, Khoo V, Escudier B, Joniau S, Bossi A, Ost P, et al. Radiotherapy for renal-cell carcinoma. Lancet Oncol. 2014;15(4):e170–7. 10.1016/S1470-2045(13)70569-2. 24694640 10.1016/S1470-2045(13)70569-2

[38] Sundaram B, Pandian N, Mall R, Wang Y, Sarkar R, Kim HJ, et al. NLRP12-PANoptosome activates PANoptosis and pathology in response to heme and PAMPs. Cell. 2023;186(13):2783-801 e20. 10.1016/j.cell.2023.05.005. 37267949 10.1016/j.cell.2023.05.005 PMC10330523

[39] Lee S, Karki R, Wang Y, Nguyen LN, Kalathur RC, Kanneganti TD. AIM2 forms a complex with pyrin and ZBP1 to drive PANoptosis and host defence. Nature. 2021;597(7876):415–9. 10.1038/s41586-021-03875-8. 34471287 10.1038/s41586-021-03875-8 PMC8603942

[40] Shen H, Geng Z, Nie X, Liu T. Erianin induces ferroptosis of renal cancer stem cells via promoting ALOX12/P53 mRNA N6-methyladenosine modification. J Cancer. 2023;14(3):367–78. 10.7150/jca.81027. 36860916 10.7150/jca.81027 PMC9969579

