# Supplementary material for: Superparamagnetic iron oxide nanoparticle-driven 3-aroyl-1,4-diarylpyrrole nanocomposites (ARDAP@SPION-PEI) mediate renal cancer cell PANoptosis by regulating chromatin accessibility
Source: Mol Biomed. 2026 May 15;7:70. doi: 10.1186/s43556-026-00470-z (PMC13179415; doi:10.1186/s43556-026-00470-z)
Supplement: Supplementary file 1 — Supplementary Material 1. [file 43556_2026_470_MOESM1_ESM.docx]

**Supplementary materials**

**Title:** Superparamagnetic iron oxide nanoparticle-driven 3-aroyl-1,4-diarylpyrrole nanocomposites (ARDAP@SPION-PEI) mediate renal cancer cell PANoptosis by regulating chromatin accessibility

**Authors:** Hongliang Shen^1,2^*^#^, Zeyu Cui^3^*, Yilun Wu^4^*, Michela Puxeddu^5^*, Yanchen Lai^3^, Ren Mo^6^, Boyu Yang^1,2^, Yinong Niu^1,2^, Yichao Wen^3^, Xiling Du^7^, Romano Silvestri^5#^, Te Liu^3^*^#^

**Author affiliations:** ^1^ Department of Urology, Beijing Friendship Hospital, Capital Medical University, Beijing 100050, China. ^2^ Institute of Urology, Beijing Municipal Health Commission, Beijing 100050, China. ^3^ Shanghai Geriatric Institute of Chinese Medicine, Shanghai University of Traditional Chinese Medicine, Shanghai 200031, China. ^4^ College of Biotechnology And Pharmaceutical Engineering, Nanjing Tech University, Nanjing 211816, China. ^5^ Department of Drug Chemistry and Technologies, Sapienza University of Rome, Rome, Italy. ^6^ Department of Urology, Inner Mongolia people’s Hospital, Inner Mongolia Urological Institute, Hohhot, Inner Mongolia 010017, China. ^7^ School of Life Science and Technology, Tongji University, Shanghai 200092, China.

* These authors contributed equally to this work and shared the first authorship.

***Corresponding author:** Hongliang Shen, Department of Urology, Beijing Friendship Hospital, Capital Medical University, 95 Yongan Road, Beijing, China, 100050, Phone: 86-10-63138585; Fax: 86-10-63138585; E-Mail: [shenhl2004@163.com](mailto:shenhl2004@163.com). Prof. Te Liu, Shanghai Geriatric Institute of Chinese Medicine, Shanghai University of Traditional Chinese Medicine, 365 South Xiangyang Road, Shanghai, China, 200031, Phone: 86-21-64720010; Fax: 86-21-64720010; E-Mail: [liute1979@shutcm.edu.cn](mailto:liute1979@shutcm.edu.cn). Prof. Romano Silvestri, Department of Drug Chemistry and Technologies, Sapienza University of Rome, Piazzale Aldo Moro 5, I-00185 Rome, Italy, 00185, Phone: 39-6-49911; Fax: 39-6-49911; E-Mail: [romano.silvestri@uniroma1.it](mailto:romano.silvestri@uniroma1.it).

**Figure S1**

**
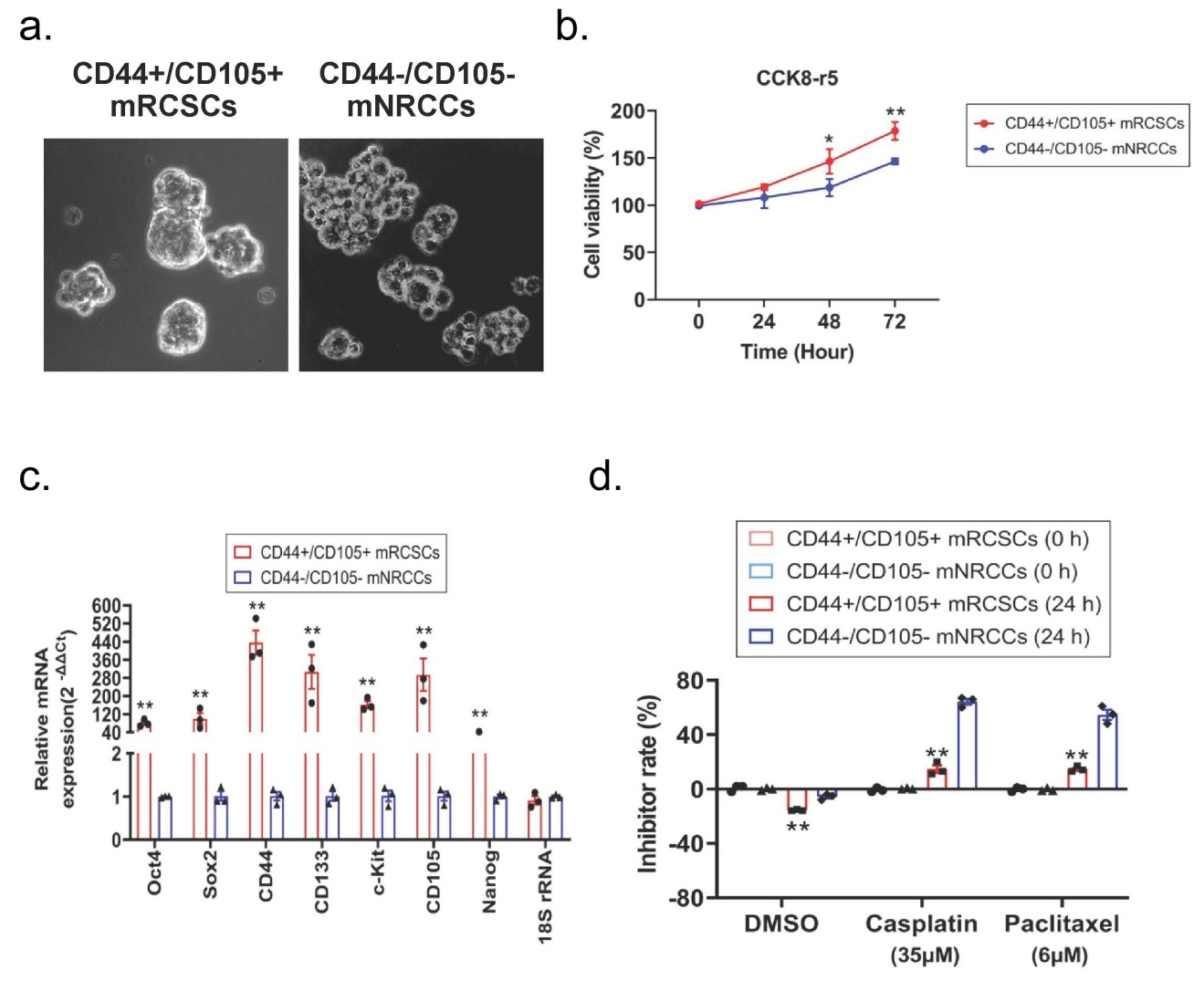
**

**Figure. S1 The stemness identification of CD44+/CD105+ mRCSCs.** (a) The CD44+/CD105+ mRCSCs form small, non-adherent, non-symmetric spheres under stem cell-selective conditions; magnification = 200×. (b) The cell viability rates of each cells were tested by CCK-8 assay; * p<0.05, ** p<0.01 vs CD44-/CD105- mNRCCs (mouse normal renal cell carcinoma cells); t test; n=3. (c) Expression levels of stem cell biomarkers in each cells were tested by qPCR; ** p<0.01 vs CD44-/CD105- mNRCCs; t test; n=3. (d) The inhibition rates of each cells treated with casplatin or paclitaxel were tested by CCK-8 assay; ** p<0.01 vs CD44-/CD105- mNRCCs; t test; n=3.

**Figure S2**

**
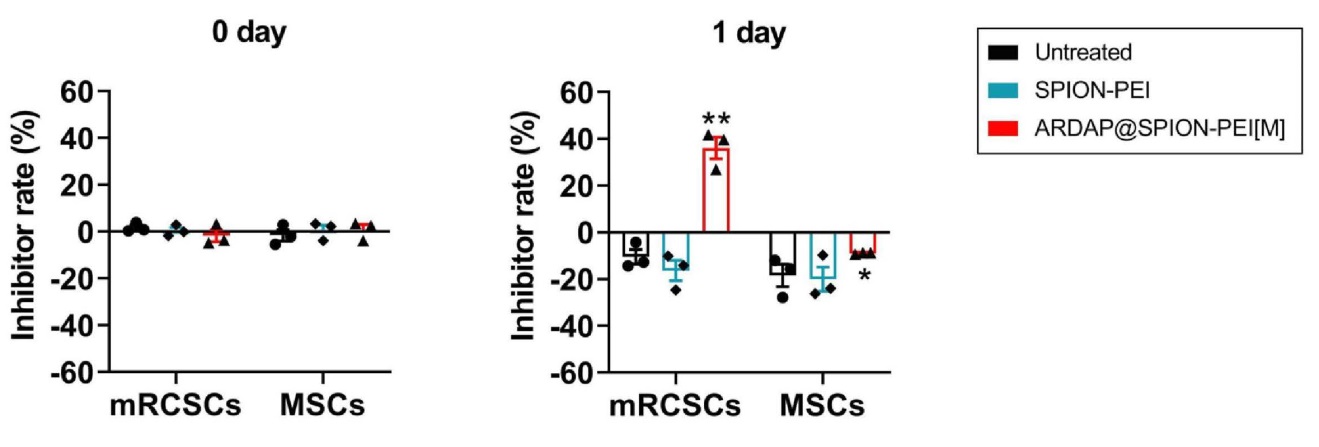
**

**Figure. S2 CCK-8 assay.** The results of CCK-8 assay showed the proliferation inhibition rate of mRCSCs after treatment with ARDAP@SPION-PEI[M] for 1 day was significantly higher than that in the SPION control group. However, ARDAP@SPION-PEI[M] did not significantly affect the normal proliferation activity of murine mesenchymal stem cells (MSCs) in vitro. *p<0.05 vs SPION-PEI; **p<0.01 vs SPION-PEI; t test; n=3.

**Figure S3**

**
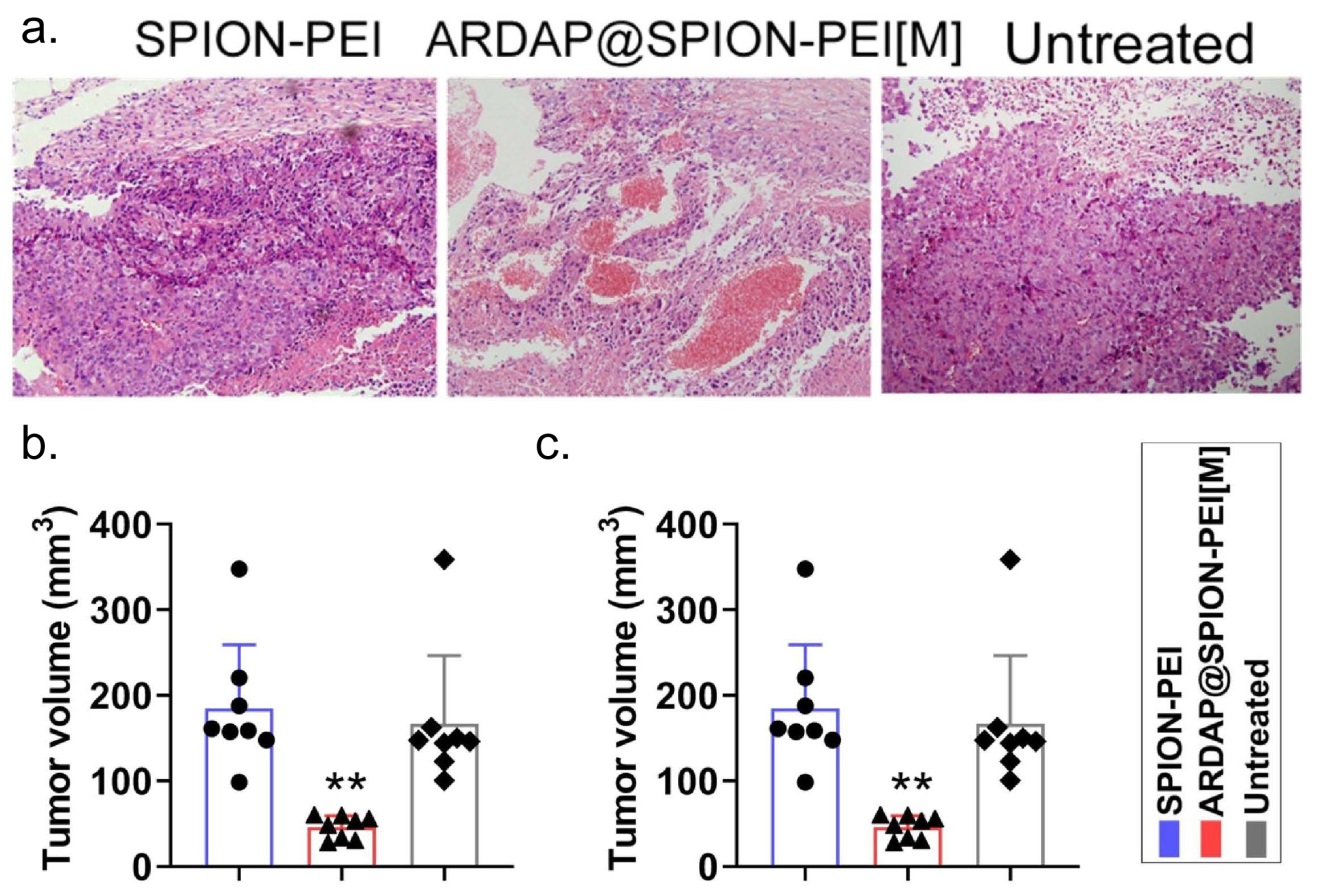
**

**Figure. S3 ARDAP@SPION-PEI exposure significantly inhibited in vivo growth in mRCSCs.** (a) The result of Hematoxylin and eosin staining on tumor tissue of three group. Magnification: 400×. (b) The tumor volumes of three group samples. **P < 0.01 vs. SPION-PEI or Untreated group, Student t test (n = 8). (c) The tumor weights of three group samples. **P < 0.01 vs. SPION-PEI or Untreated group, Student t test (n = 8).

**Figure S4**

**
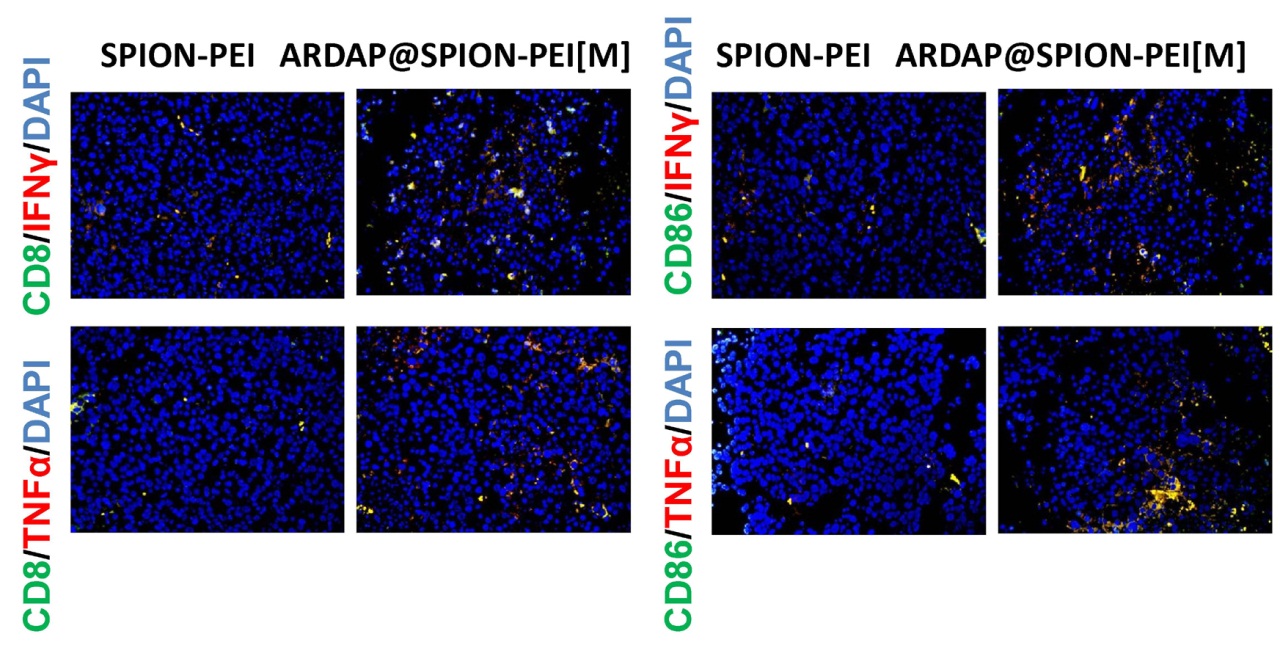
**

**Figure. S4 The results of immunofluorescence staining assay.** The results shown that cytotoxic T cells biomarkers (CD8+/IFNγ+ or CD8+/ TNFα+) protein expression levels were significantly higher in the tumor tissues of ARDAP@SPION-PEI-treated mice than in those of control group. Meanwhile, The type 1 macrophage (Mφ 1) biomarkers (CD86+/INFγ+ or CD86+/ TNFα+) protein expression levels were also significantly higher in the tumor tissues of ARDAP@SPION-PEI-treated mice than in those of control group. Magnification: 400×.

**Figure S5**

**
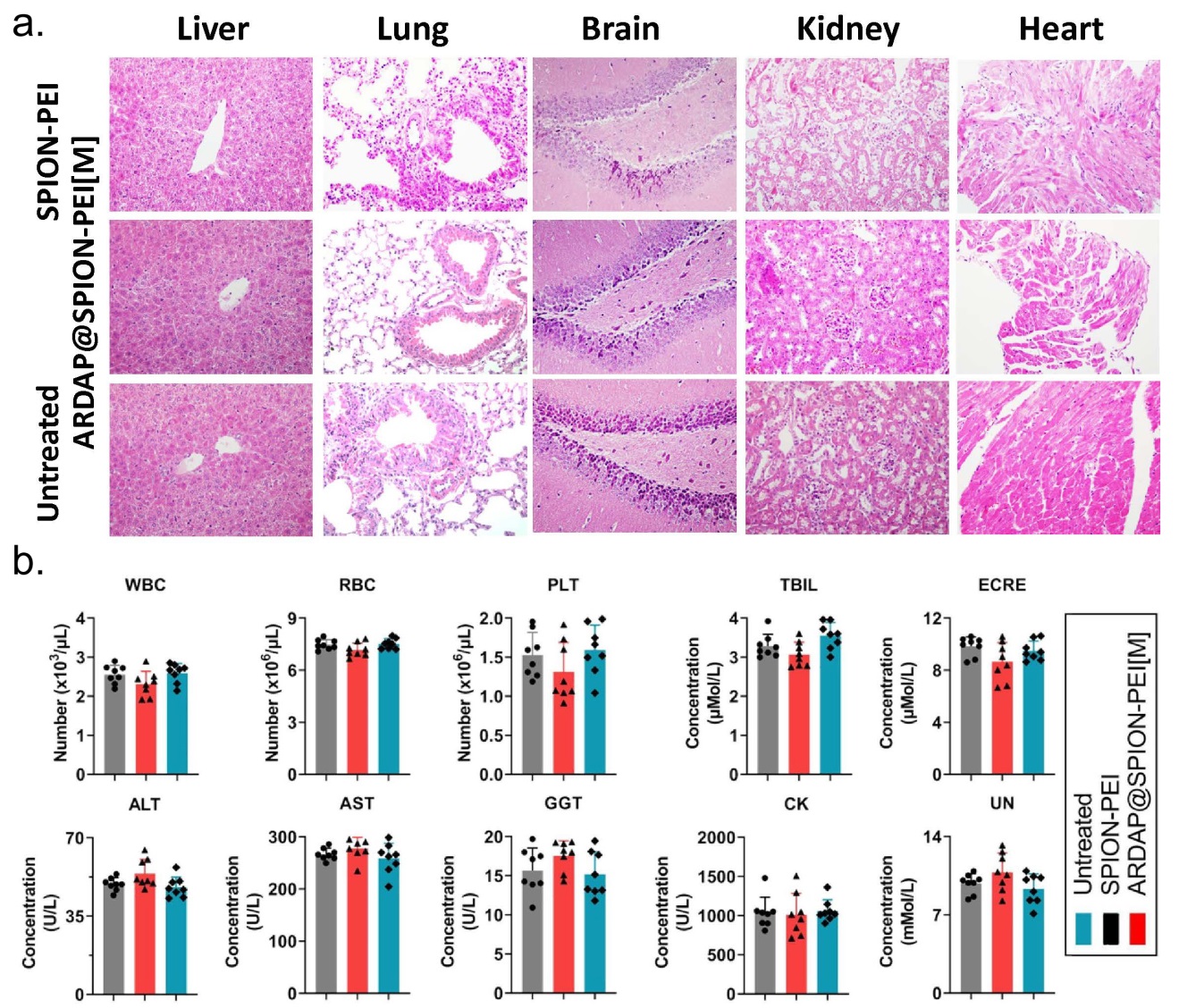
**

**Figure. S5 In vivo safety testing of drugs.** (a) The results of H&E staining of pathological examinations. Magnification: 400×. (b) Peripheral blood index detection of liver, kidney, and heart function.

**Figure S6**

**
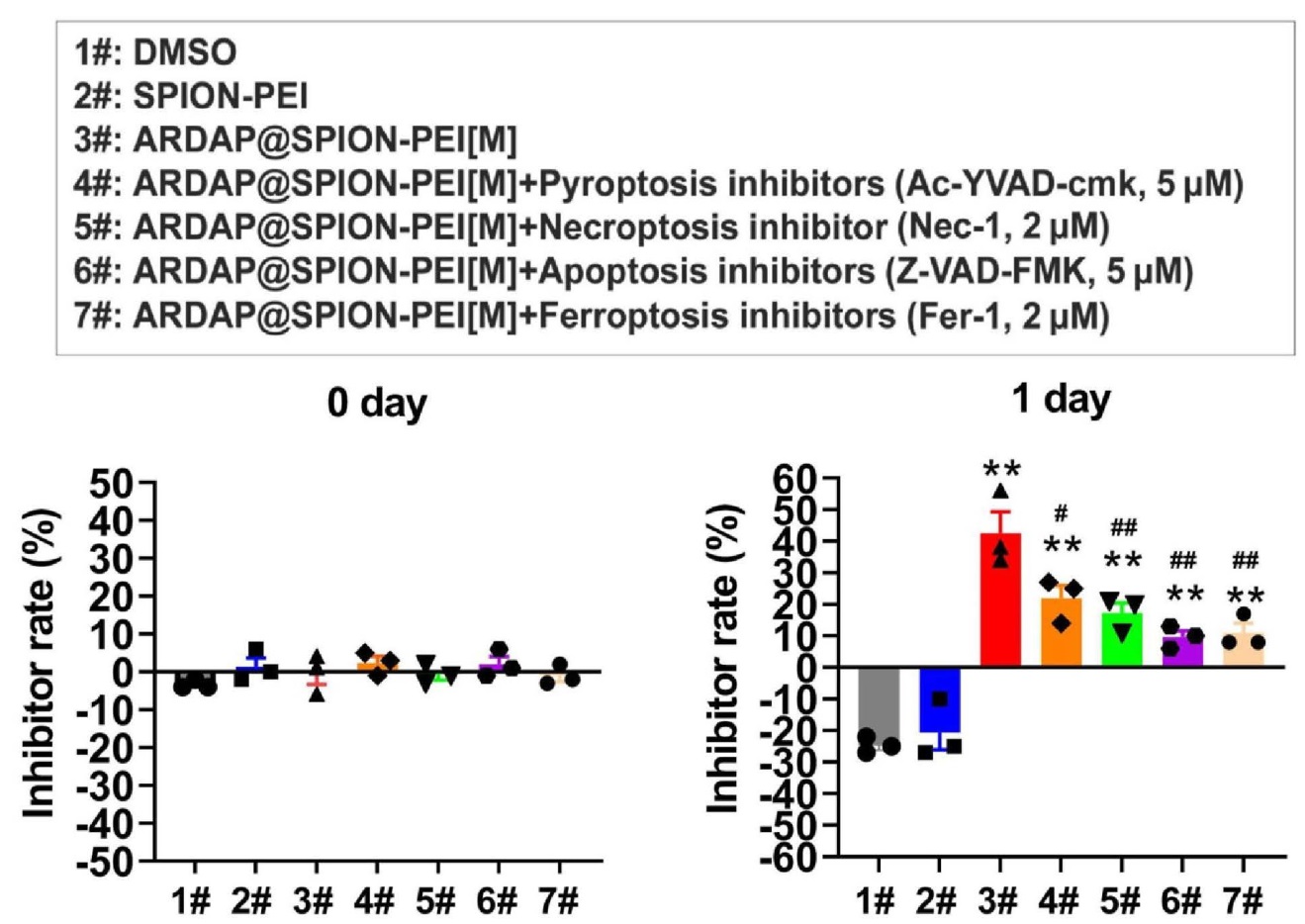
**

**Figure. S6 CCK-8 detection for the rescued effect of multiple inhibitors.** The mRCSCs were respectively treated with specific inhibitors of pyroptosis/apoptosis/necroptosis/ferroptosis in combination with ARDAP@SPION-PEI[M], and the proliferation inhibition rate was measured using CCK-8 assay. The results of CCK-8 assay revealed that any combination of the above inhibitors with ARDAP@SPION-PEI[M] treatment could significantly reduce the proliferation inhibition rate of mRCSCs. **p<0.01 vs SPION-PEI; ^##^p<0.01 vs ARDAP@SPION-PEI[M]; ^#^p<0.05 vs ARDAP@SPION-PEI[M]; t test; n=3.

**Figure S7**

**
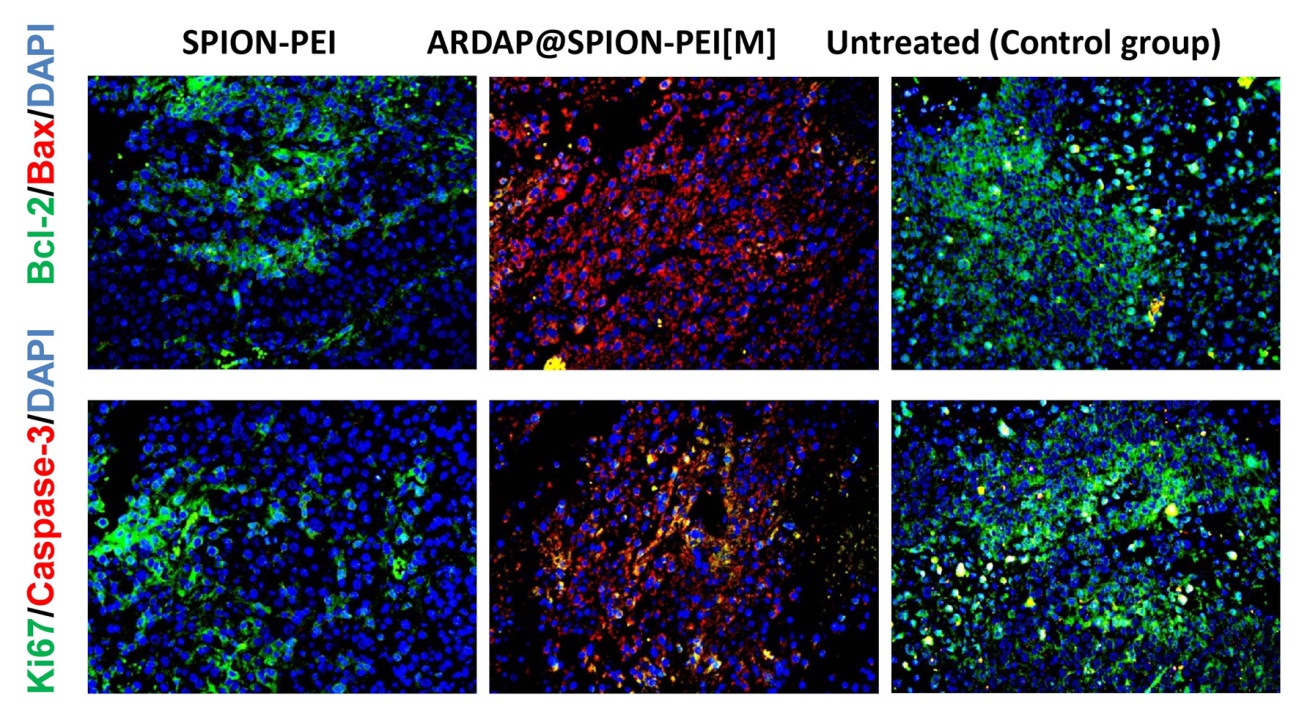
**

**Figure. S7 The results of immunofluorescence staining assay.** The results shown that Ki67 and Bcl-2 protein expression levels were significantly lower in the tumor tissues of ARDAP@SPION-PEI-treated mice than in those of control group (Untreated group) and SPION-PEI-treated mice. However, the Caspase-3 and Bax protein expression levels were inversely related to the aforementioned trend. Magnification: 400×.

**Figure S8**

**
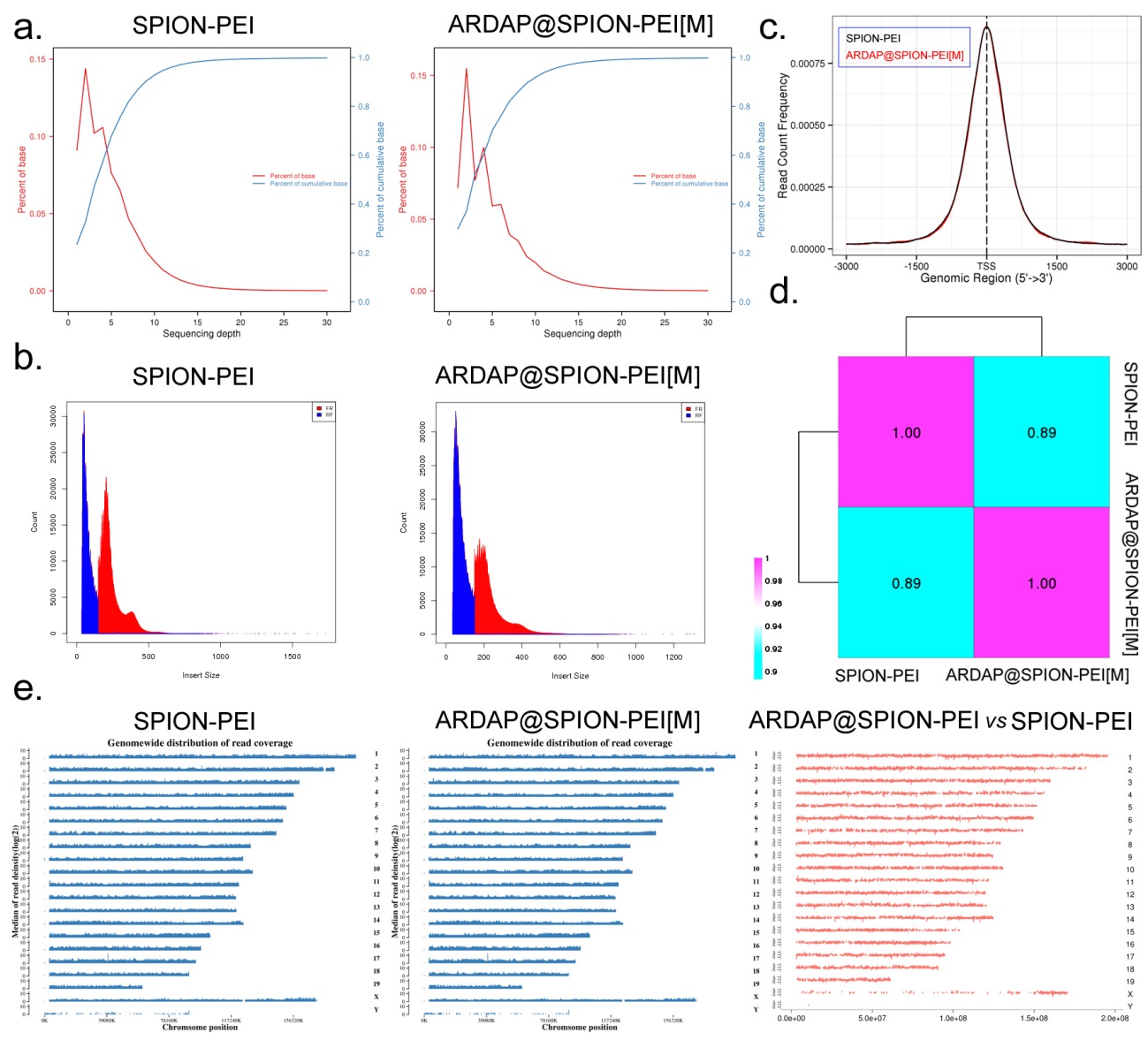
**

**Figure. S8 Our ATAC-seq data were high-quality.** (a) Base coverage depth distribution curve and coverage distribution curve of unique map reads. (b) Insertion fragment lengths. (c) Statistical results of the average peak number in the TSS region of the genome. (d) Sliding window and clustering heatmap combined for calculating read abundance across the entire genome. (e) Distribution map of base chromosome coverage for unique map reads.

**Figure S9**

**
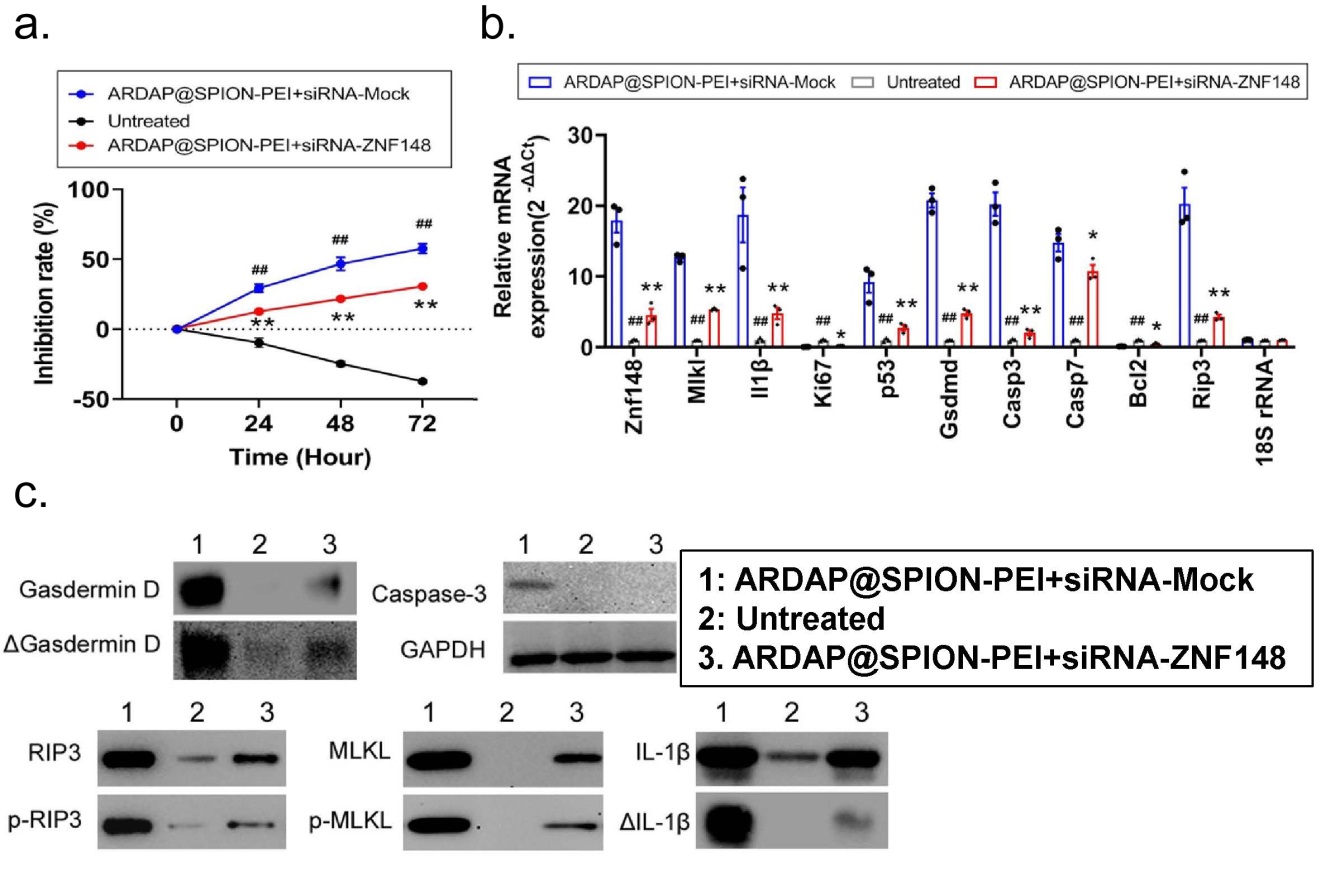
**

**Figure. S9 To interference the expression of ZNF148 by siRNA attenuated expression of ARDAP@SPION-PEI-mediated PANoptosis key genes in mRCSCs.** (a) The results of CCK-8 assay. ##P < 0.01 vs. Untreated, **P < 0.01 vs. ARDAP@SPION-PEI+siRNA-Mock, Student t test (n = 3). (b) The results of qPCR test. ##P < 0.01 vs. Untreated, **P < 0.01 vs. ARDAP@SPION-PEI+siRNA-Mock, *P < 0.05 vs. ARDAP@SPION-PEI+siRNA-Mock, Student t test (n = 3). (c) The results of Western blotting assay.

**Figure S10**

**
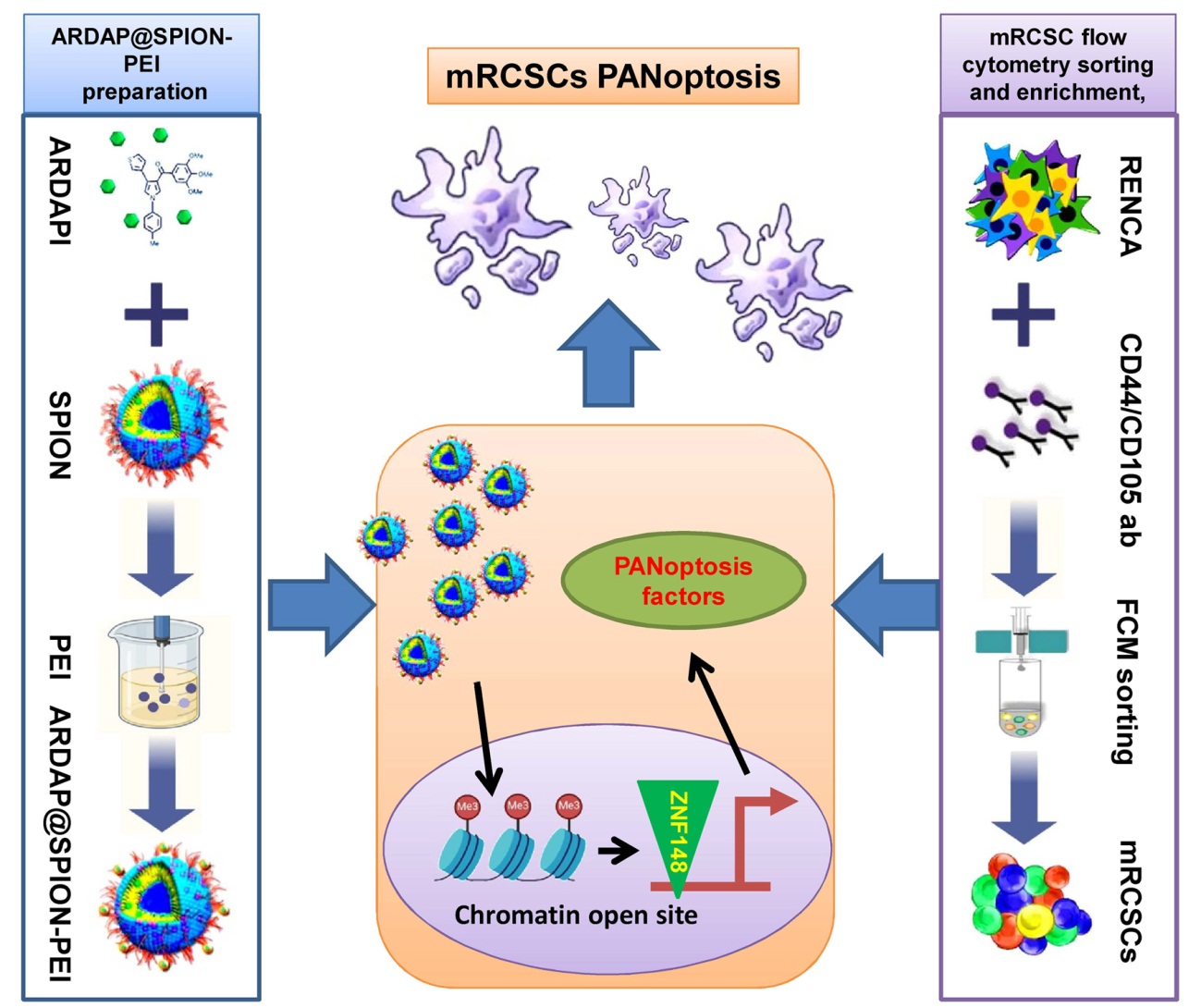
**

**Figure. S10 ARDAP@SPION-PEI increased ZNF148 expression by regulating the chromatin structure and accessibility and finally activating key PANoptosis pathway gene transcription in mRCSCs.**

**Materials and Methods**

***ARDAP@SPION-PEI preparation***

SPION-PEI (100 mg; Novobio, Shanghai, China) were mixed with 100  mg of ARDAP in 1.0  mL of phosphate buffer (0.2 mol/L Na_2_HPO_4_, pH 6.0) through ultrasonication for 60 min. ARDAP@SPION-PEI were separated from the mixture by using a neodymium magnet, washed with water and ethanol three times, and finally, vacuum-dried at room temperature.

***Nanoparticle tracking analysis***

By using a dark-field microscope (NS500; Nanosight, Amesbury, UK) equipped with a 45-mW 405-nm laser and an electron multiplying charge-coupled device, we tracked the Brownian motion of SPION single particles.

***Transmission electron microscopy***

Tissue samples were fixed in 1% glutaraldehyde (Sigma-Aldrich, St. Louis, USA) for 4 h and then in 1% osmium acid for 1 h. After dehydration in acetone, they were embedded in resin 12 (Ted Pella, USA). Ultrathin sections (cross-section thickness = 70 nm) were mounted on a copper mesh, stained with 1% uranyl acetate (Sigma-Aldrich) and 1% lead citrate (Sigma-Aldrich), and observed and photographed under the JEM-1230 transmission electron microscope (JEOL, Japan).

***Cell culture and intervention***

The mouse renal cancer cell line RENCA was purchased from the Oricell, Cyagen Biosciences (Suzhou) Inc. These cells were cultured in Dulbecco’s modified Eagle medium (DMEM) containing 10% fetal bovine serum and 1% penicillin–streptomycin at 37 °C under 5% CO_2_. Only log-phase cells were selected for further analysis. During analysis, experimental and control group cells were exposed to 40 nM ARDAP@SPION-PEI (hereafter, denoted as ARDAP@SPION-PEI[M]) and SPION-PEI for 24 h, respectively [1, 2].

***mRCSC flow cytometry sorting, enrichment, and intervention***

As reported previously [3, 4], 2 × 10^8^/mL RENCA cells were mixed with 0.5 mL of ice-cold sterile phosphate-buffered saline (PBS; HyClone), followed by exposure to 5 μL of antimouse CD105-FITC (Millenyi) and 5 μL of antimouse CD44-PE (Millenyi) antibodies. The final reaction concentration was 0.01 mg/mL. The mixture was incubated at 4°C for 30 min in the dark. Thereafter, the cells were washed twice with ice-cold PBS, and then mouse RCSCs (mRCSCs) were isolated and enriched through flow cytometry on BD FACS Aria (BD Bioscience, CA, USA). The cell count was adjusted to 1,000/mL and inoculated into nonadult spherical clusters. The cells were then cultured in DMEM–F12 medium (HyClone) containing 10 ng/mL basic fibroblast growth factor, 10 ng/mL epidermal growth factor, 5 μg/mL insulin, and 0.5% bovine serum albumin (all from Sigma-Aldrich) until the third generation. These cells were divided into medium-dose experimental, high-dose experimental, and control groups and exposed to ARDAP@SPION-PEI[M], 80 nM ARDAP@SPION-PEI (hereafter, denoted as ARDAP@SPION-PEI[H]), and SPION-PEI (at equivalent concentrations), respectively. Blank group cells were not subjected to any drug. In all groups, drug exposure was allowed for 24 h.

***Cell counting kit 8 assay***

Cells from each group were inoculated into a 96-well plate at 1,000 cells/well and incubated for 24 h. To each well, we added 10 μL of cell counting kit 8 (CCK-8, final concentration of 10%) reagent (Beyotime, Zhejiang, China) and incubated for 2 h. The absorbance at 450 nm of the developed color was measured on an enzyme-linked immunosorbent assay reader. The cell proliferation inhibition rate (%) was calculated as [1 – (absorbance at 450 nm of experimental group cells/absorbance at 450 nm of control group cells)] × 100.

***Propidium iodide staining and flow cytometry identification***

mRCSCs (2 × 10^5^/mL) were fixed in 1 mL of 70% ice-cold ethanol for 48 h. Next, the cells were centrifuged at 1,500 rpm at 4°C for 5 min. The cell pellets were collected and stained using a propidium iodide staining solution (final concentration of 100ug/ml, Sigma Chemicals) at 4°C for 30 min in the dark. Cell-cycle distribution was assessed through flow cytometry (BD FACS Aria), and the data were analyzed using CellQuest.

***Transwell assay***

We inoculated 1 × 10^5^ cells in the upper chamber of a transwell chamber, added 400 μL of culture medium containing 10% fetal bovine serum to its lower chamber, and incubated it for 24 h. Next, the cells were fixed with 4% paraformaldehyde (Sigma-Aldrich) at room temperature for 20 min and stained with 0.1% crystal violet for 10 min. Finally, the total number of migrated cells in three nonoverlapping fields was counted under a microscope.

***Assessment of effects of ARDAP@SPION-PEI on zebrafish embryo vascular development***

In total, 30 zebrafish embryos (24 h post fertilization; strain: Kdrl::mCherry; Shanghai Research Centre for Model Organisms, Shanghai, China) were added to each well of a six-well plate, which also contained E3 embryonic culture medium (0.58 g/L NaCl, 0.27 g/L KCl, 0.97 g/L CaCl_2_·2H_2_O, 0.16 g/L MgCl_2_·6H_2_O, 1% methylene blue; pH 7.2; all from Sigma-Aldrich; Merck KGaA). Next, SPION-PEI (40 nM), ARDAP@SPION-PEI[M] (40 nM), or ARDAP@SPION-PEI[H] (80 nM) were added according to group allocation[1, 2]. After 12 h of treatment, the medium was refreshed, and the phenotype changes in zebrafish embryos were assessed under a Nikon SMZ 1500 stereomicroscope (Nikon; magnification: 40×). In particular, the numbers of fully developed intersegmental blood vessels (ISVs) and dorsal longitudinal anastomotic vessels (DLAVs) in each embryo were counted. The angiogenesis inhibition rate was calculated as follows (24): [1 − (average number of ISVs in the embryoid body in the experimental group/average numbers of ISVs and DLAVs in the embryoid body in the control group)] × 100.

***In vivo xenograft experiments***

Next, 1 × 10^6^/mL log-phase cells were inoculated subcutaneously on the backs of male Balb/c mice (age = 6–8 weeks old), provided by the Experimental Animal Center of Shanghai University of Traditional Chinese Medicine. When the tumors grew to a diameter of approximately 2 mm, we randomly divided the tumor-bearing mice into two groups (n = 8 per group). All mice were intraperitoneally injected with 100 μL of 25 mg/kg ARDAP@SPION-PEI[M] or SPION-PEI once every 2 days[1, 2]. After 30 consecutive days of intervention, the mice were euthanized, and their tumors were removed and weighed. Finally, the tumor volume (mm^3^) was calculated as (a × b × b)/2, where *a* and *b* denote the longest and shortest axes (both in mm). All animal experiments were performed in accordance with the guidelines of the NIH for the care and use of laboratory animals. The study protocol was approved by the Committee on the Use of Live Animals in Teaching and Research, Shanghai Geriatric Institute of Chinese Medicine, Shanghai, China (SHAGE20220908).

***Hematoxylin and eosin (H&E) staining***

Tissue samples were fixed in 4% paraformaldehyde, dehydrated, and embedded in paraffin. The tissue paraffin blocks were then sliced into 4-μm-thick sections on a microtome and placed on glass slides. This was followed by dewaxing in xylene and ethanol gradient dehydration. The slides were then stained with a hematoxylin staining solution at room temperature for 5 min, differentiated using 1% hydrochloric acid ethanol for 30 s, exposed to dilute ammonia water for 1 min to develop the blue color, and washed with distilled water for 5 min. Next, the slides were stained with an eosin staining solution at room temperature for 2 min, washed with distilled water for 2 min, subjected to ethanol gradient decolorization, and permeated with xylene for 2 min. Finally, all slides were sealed with neutral gum.

***Immunohistochemical staining assay***

Fresh tissue samples were fixed in 4% paraformaldehyde at room temperature for 30 min, followed by ethanol gradient dehydration, paraffin embedding, slicing in 6-μm-thick sections, and dewaxing in xylene. All tissue sections were sealed with an immunohistochemistry blocking solution (Beyotime) at 37°C for 30 min. Next, the blocking solution was discarded, and the slides were cleaned three times with an immunohistochemistry cleaning solution (Beyotime) at room temperature for 5 min each time. Next, the slides were incubated with primary antibodies (MLKL Mouse Antibody (3B2)(sc-293201), Phospho-MLKL (Ser345) (D6E3G) Rabbit Monoclonal Antibody (#37333), Gasdermin D Mouse Antibody (H-6) (sc-376318), Cleaved Gasdermin D (Asp276) (E3E3P) Rabbit Monoclonal Antibody (#10137), RIP3 Mouse Antibody (B-2) (sc-374639), Phospho-RIP3 (Thr231/Ser232) (E7S1R) Rabbit Monoclonal Antibody (#91702), IL-1 beta/IL1B Mouse Antibody (AS57) (sc-52771), Cleaved-IL-1 beta (Asp117) (E7V2A) Rabbit Monoclonal Antibody (#63124), Cell Signaling Technology, Inc, USA, Santa Cruz Biotechnology, Inc, USA) at 37°C for 45 min, followed by three washings with the immunohistochemistry cleaning solution at room temperature for 5 min each time. The slides were then incubated with secondary antibodies (Anti-mouse IgG (H+L), F(ab')2 Fragment (Alexa Fluor® 488 Conjugate) (#4408), Anti-rabbit IgG (H+L), F(ab')2 Fragment (Alexa Fluor® 555 Conjugate)(#4413), Cell Signaling Technology, Inc, USA) at 37°C for 45 min, followed by three washings with the immunohistochemistry cleaning solution at room temperature for 5 min each time. Finally, we sealed slides using a DAPI-containing immunofluorescence blocking solution (Sigma-Aldrich).

***RNA extraction and reverse transcription quantitative polymerase chain reaction***

To extract RNA from the tissues and cells, we used the RNAprep pure Tissue Kit (Tiangen Biotech, Beijing, China), according to the manufacturer’s instructions. In brief, cells were treated with 800 μL of lysis solution, followed by vigorous shaking. Next, 200 μL of chloroform was added, followed by gentle mixing with inversion and centrifugation at 13,400 ×g at 4°C for 15 min. The supernatant was collected and added to two volumes of anhydrous ethanol, followed by gentle mixing with inversion and centrifugation at 13,400 ×g at 4°C for 30 min. The RNA-containing precipitate was resuspended in 500 μL of 75% ethanol and centrifuged at 13,400 ×g at 4°C for 15 min. Next, the precipitate was thoroughly dissolved in 300 μL of DECP water. Finally, 1 μL of this mixture was used to detect the total RNA concentration, as well as the RNA purity based on the OD260/OD280 ratio (pure RNA OD260/OD280 ratio range = 1.8–2.0). Next, we used Tiangen Biotech’s mRNA first-strand cDNA kit, according to the manufacturer’s instructions. In brief, 20 μL of total RNA (100 ng/μL), 25 μL of 2× mRNA RT Reaction Buffer, 4 µL of 1× mRNA RT Enzyme Mix, and 6 μL of RNase-free deionized water were mixed thoroughly. Next, the mRNA was reverse transcribed into cDNA using reverse transcriptase plus A-tail at 42°C for 60 min in a quantitative polymerase chain reaction (qPCR) instrument. Finally, the enzyme was inactivated at 95°C for 3 min. For qPCR, the reaction volume included the following: 10 μL of 2× mRcute PlusmRNAPremix (with SYBR), 1 μL each of 1× 10 μM forward and reverse primer, 4 μL of the prepared first-strand cDNA, and 4 μL of deionized water. The following conditions were used for real-time fluorescence qPCR: 95°C for 15 min, followed by 40 cycles of 94°C for 20 s and 60°C for 34 s. Fluorescence values were collected after each cycle. Next, we used the 2^−ΔΔCt^ method to determine the relative expression level of genes; here, ΔCt = Ct_genes − Ct_18srRNA, and ΔΔCt = ΔCt_all_groups − ΔCt_control_group. mRNA expression levels were corrected on the basis of the 18s rRNA expression level. The details of PCR primer sequences were as follows: PCR Primer sequences information:

MLKL-FP: 5'-AATTGTACTCTGGGAAATTGCCA-3'; MLKL-RP: 5'-TCTCCAAGATTCCGTCCACAG-3'; IL1B-FP: 5'-GAAATGCCACCTTTTGACAGTG-3'; IL1B-RP: 5'-TGGATGCTCTCATCAGGACAG-3'; Ki67-FP: 5'-CAAGGCGAGCCTCAAGAGATA-3';

Ki67-RP: 5'-TGTGCTGTTCTACATGCCCTG-3'; P16-FP: 5'-CGCAGGTTCTTGGTCACTGT-3'; P16-RP: 5'-TGTTCACGAAAGCCAGAGCG-3'; P21-FP: 5'-CCTGGTGATGTCCGACCTG-3'; P21-RP: 5'-CCATGAGCGCATCGCAATC-3';

P53-FP: 5'-GCGTAAACGCTTCGAGATGTT-3'; P53-RP: 5'-TTTTTATGGCGGGAAGTAGACTG-3'; GSDMD-FP: 5'-CCATCGGCCTTTGAGAAAGTG-3'; GSDMD-RP: 5'-ACACATGAATAACGGGGTTTCC-3'; Casp3-FP: 5'-ATGGAGAACAACAAAACCTCAGT-3'; Casp3-RP: 5'-TTGCTCCCATGTATGGTCTTTAC-3';

Casp9-FP: 5'-TCCTGGTACATCGAGACCTTG-3'; Casp9-RP: 5'-AAGTCCCTTTCGCAGAAACAG-3'; Casp7-FP: 5'-CGGAATGGGACGGACAAAGAT-3'; Casp7-RP: 5'-CTTTCCCGTAAATCAGGTCCTC-3'; BCL2-FP: 5'-GTCGCTACCGTCGTGACTTC-3';

BCL2-RP: 5'-CAGACATGCACCTACCCAGC-3'; BAX-FP: 5'-TGAAGACAGGGGCCTTTTTG-3'; BAX-RP: 5'-AATTCGCCGGAGACACTCG-3'; XIAP-FP: 5'-CGAGCTGGGTTTCTTTATACCG-3'; XIAP-RP: 5'-GCAATTTGGGGATATTCTCCTGT-3'; RREB1-FP: 5'-CCCACTAAGATGTGACATTTGCT-3'; RREB1-RP: 5'-GCAGGAATCGAAGGGTTGTTCT-3'; MAZ-FP: 5'-GCCCCAGTTGCATCTGTCTT-3'; MAZ-RP: 5'-CTTCGGAGGTTGTAGCCGTT-3'; RIP3-FP: 5'-TGGGCCTGCTAAGATGGCT-3'; RIP3-RP: 5'-CTGCCAGAGTGTGGATTTGGT-3'; 18SrRNA-FP: 5'-AGGGGAGAGCGGGTAAGAGA-3'; 18SrRNA-RP: 5'-GGACAGGACTAGGCGGAACA-3'.

***Protein extraction and Western blotting assay***

Cells from each group were lysed using a 2 × loading lysis buffer (50 mM Tris–HCl, pH 6.8, 2 % sodium dodecyl sulfate, 10 % β-mercaptoethanol, 10 % glycerol and 0.002 % bromophenol blue) to extra the total protein. The total protein in cells from each group was separated through 12% sodium dodecyl sulfate polyacrylamide gel electrophoresis and then transferred onto polyvinylidene difluoride (PVDF) membranes (Millipore). After sealing and washing the membranes, we incubated the membranes with the primary antibodies (Mouse Reactive PANoptosis Antibody Sampler Kit (#70934), GAPDH (14C10) Rabbit Monoclonal Antibody (#2118), Cell Signaling Technology, Inc, USA) at 37°C for 45 min, followed by thorough washing. Next, the membranes were incubated with the secondary antibodies (Anti-rabbit IgG, HRP-linked Antibody (#7074), Cell Signaling Technology, Inc, USA) at 37°C for 45 min, followed by four washings with tris-buffered saline with Tween 20 at room temperature for 14 min each time. Finally, immunoreactive protein bands were revealed using an enhanced chemiluminescence kit (Pierce Biotechnology, Sigma-Aldrich).

***Assay for Transposase-Accessible Chromatin-seq (ATAC-seq)***

ATAC-seq, including sample preparation experiments and sequencing experiments, was performed according to the standard protocol provided by Illumina [5] at Company Biomarker (Beijing, China). In brief, single-cell suspensions were prepared, and their nuclei were extracted. Subsequently, the Tn5 transposon reaction was performed, followed by purification, PCR, and sequencing of the reaction products. Finally, the raw reads obtained from ATAC-seq were subjected to low-quality splicing and filtering to obtain clean reads. All clean reads were aligned with reference genome sequences to obtain information on the alignment locations. The peak positions were obtained by comparing the position information of the reads on the genome, and the peak positions of the genome were annotated to obtain a list of genes affected by the peak. Finally, motif analysis was performed on the obtained peaks, and the potential transcription factor binding site information was acquired.

***Chromatin immunoprecipitation–PCR assay (ChIP-PCR)***

Cells were fixed with 1% paraformaldehyde at 37°C for 30 min and then incubated with 125 mM glycine at room temperature for 10 min to remove crosslinking. Next, the cells were exposed to the chromatin immunoprecipitation (ChIP) cell lysis solution (Beyotime), followed by sonication on ice until DNA broke into 200–1,000-bp chromatin fragments. This was followed by incubation with rabbit antimouse histone H3 (tri methyl K4) antibody [EPR20551-225] (Abcam) at 4°C overnight. The DNA was then subjected to proteinA/G plus-agarose adsorption to obtain an immunoprecipitate. The treated DNA was subjected to the following PCR conditions: 33 cycles of denaturing at 95°C for 30 s, followed by annealing at 55°C for 30 s and extension at 72°C for 30 s. The amplification products were visualized through agarose gel electrophoresis.

***Luciferase reporter assay***

Here, the luciferase reporter assays were performed, as described previously [3]. HeLa cells were seeded in a 48-well plate at 3 × 10^4^/well and cotransfected with 20 ng of psiCHECK-1#Motif-WT-Znf148 (insert fragment: 5′-…GGAGGCGG…-3′), psiCHECK-2#Motif-WT-Znf148 (insert fragment: 5′-…GCAGGCGG…-3′), psiCHECK-Motif-Mut-Znf148 (insert fragment: 5′-…atcattac…-3′), or psiCHECK-Blank (Novobio) by using Lipofectamine 2000, according to the manufacturer’s instructions. After 48 h, luciferase activity was measured using the Dual-Luciferase Reporter Assay System (Promega, Madison, USA).

***Statistical analysis***

Each experiment was performed at least three times. Between-group differences in all data, presented as means and the standard errors, were evaluated using the Student *t* test. *P* < 0.05 was considered to indicate statistical significance.

**References:**

1. La Regina G, Bai R, Coluccia A, Famiglini V, Passacantilli S, Naccarato V, et al. 3-Aroyl-1,4-diarylpyrroles Inhibit Chronic Myeloid Leukemia Cell Growth through an Interaction with Tubulin. ACS Med Chem Lett. 2017; 8: 521-6.

2. Puxeddu M, Shen H, Bai R, Coluccia A, Nalli M, Mazzoccoli C, et al. Structure-activity relationship studies and in vitro and in vivo anticancer activity of novel 3-aroyl-1,4-diarylpyrroles against solid tumors and hematological malignancies. Eur J Med Chem. 2020; 185: 111828.

3. Si Y, Liu J, Shen H, Zhang C, Wu Y, Huang Y, et al. Fisetin decreases TET1 activity and CCNY/CDK16 promoter 5hmC levels to inhibit the proliferation and invasion of renal cancer stem cell. J Cell Mol Med. 2019; 23: 1095-105.

4. Shen H, Geng Z, Nie X, Liu T. Erianin Induces Ferroptosis of Renal Cancer Stem Cells via Promoting ALOX12/P53 mRNA N6-methyladenosine Modification. J Cancer. 2023; 14: 367-78.

5. Grandi FC, Modi H, Kampman L, Corces MR. Chromatin accessibility profiling by ATAC-seq. Nat Protoc. 2022; 17: 1518-52.
